# Steady-state memory-phenotype conventional CD4^+^ T cells exacerbate autoimmune neuroinflammation in a bystander manner via the Bhlhe40/GM-CSF axis

**DOI:** 10.1038/s12276-023-00995-1

**Published:** 2023-05-01

**Authors:** Min-Ji Cho, Hong-Gyun Lee, Jae-Won Yoon, Gil-Ran Kim, Ja-Hyun Koo, Reshma Taneja, Brian T. Edelson, You Jeong Lee, Je-Min Choi

**Affiliations:** 1 Department of Life Science, College of Natural Sciences, Hanyang University, Seoul, 04763 Republic of Korea; 2 Ann Romney Center for Neurologic Diseases, Brigham and Women’s Hospital, Harvard Medical School, Boston, MA 02115 USA; 3 The Ragon Institute of Massachusetts General Hospital, Massachusetts Institute of Technology and Harvard University, Cambridge, MA 02139 USA; 4 Department of Physiology and Healthy Longevity Translation Research Program, Yong Loo Lin School of Medicine, National University of Singapore, 117593 Singapore, Singapore; 5 Department of Pathology and Immunology, Division of Laboratory and Genomic Medicine, Washington University School of Medicine, St. Louis, MO 63119 USA; 6 Research Institute of Pharmaceutical Sciences, Seoul National University, Seoul, Republic of Korea; 7 Research Institute for Natural Sciences, Hanyang University, Seoul, 04763 Republic of Korea; 8 Research Institute for Convergence of Basic Sciences, Hanyang University, Seoul, 04763 Republic of Korea; 9 Hanyang Institute of Bioscience and Biotechnology, Hanyang University, Seoul, 04763 Korea

**Keywords:** Autoimmunity, Neuroimmunology, Lymphocyte activation

## Abstract

Memory-phenotype (MP) CD4^+^ T cells are a substantial population of conventional T cells that exist in steady-state mice, yet their immunological roles in autoimmune disease remain unclear. In this work, we unveil a unique phenotype of MP CD4^+^ T cells determined by analyzing single-cell transcriptomic data and T cell receptor (TCR) repertoires. We found that steady-state MP CD4^+^ T cells in the spleen were composed of heterogeneous effector subpopulations and existed regardless of germ and food antigen exposure. Distinct subpopulations of MP CD4^+^ T cells were specifically activated by IL-1 family cytokines and STAT activators, revealing that the cells exerted TCR-independent bystander effector functions similar to innate lymphoid cells. In particular, CCR6^high^ subpopulation of MP CD4^+^ T cells were major responders to IL-23 and IL-1β without MOG_35-55_ antigen reactivity, which gave them pathogenic Th17 characteristics and allowed them to contribute to autoimmune encephalomyelitis. We identified that Bhlhe40 in CCR6^high^ MP CD4^+^ T cells as a key regulator of GM-CSF expression through IL-23 and IL-1β signaling, contributing to central nervous system (CNS) pathology in experimental autoimmune encephalomyelitis. Collectively, our findings reveal the clearly distinct effector-like heterogeneity of MP CD4^+^ T cells in the steady state and indicate that CCR6^high^ MP CD4^+^ T cells exacerbate autoimmune neuroinflammation via the Bhlhe40/GM-CSF axis in a bystander manner.

## Autoimmunity: Bystander T cells fuel inflammation in the CNS

An antigen independent function of T cells, known as bystander-activated T cell, contributes to autoimmune disease in the CNS (Central Nervous System), and could be targeted therapeutically to help suppress neuroinflammation. A team led by Je-Min Choi from Hanyang University in Seoul, South Korea, showed in mice that different subgroups of memory-phenotype T cells naturally arising without immunization. These cells become activated in response to different immune-stimulating cytokines, even without antigen-specific immune recognition. The researchers identified one particular subgroup that is a key driver of inflammatory signaling and works with other T cells to exacerbate an experimental model of multiple sclerosis. The findings provide important insights into potential therapeutic approaches that drugs designed to block bystander-activated T cells may offer relief for people with immune-inflammatory diseases of the central nervous system.

## Introduction

Immunological memory established by antigen-specific T cells enables faster and more potent responses upon re-exposure to a previously encountered antigen, providing long-lasting immunity^1^. Although a substantial population of memory-phenotype (MP) conventional T cells exists in steady-state mice unexposed to foreign antigens, it has been reported that MP T cells are formed before birth in humans and exist in germ-free (GF) and antigen-free (AF) conditioned mice^2–6^.

After undergoing homeostatic proliferation in a lymphopenic environment, naïve T cells acquire phenotypical, functional, and gene expression features of antigen-specific memory and become MP T cells^7–10^. T cell receptor (TCR) and CD28 signaling seem to be required for the conversion of naïve CD4^+^ T cells into self-derived MP T cells in lympho-sufficient conditions. These MP CD4^+^ T cells express CD5, a marker with high affinity for self-antigens, and resist infection by mediating a Th1-like immune response without antigen stimulation^11^. A previous study confirmed that antigen-nonspecific MP CD4^+^ T cells proliferated more than lymphocytic choriomeningitis virus (LCMV)-specific T cells in an LCMV infection model. Indeed, treatment with an anti-MHCII antibody did not inhibit the proliferation of MP CD4^+^ T cells, suggesting that they play a bystander role against infection^12^.

Unlike MP CD4^+^ T cells, MP CD8^+^ T cells have been well studied to characterize their antigen-specific and bystander functions. MP CD8^+^ T cells can rapidly produce IFN-γ upon stimulation with IL-12 and IL-18 and without cognate antigen stimulation^13,14^. In particular, MP CD8^+^ T cells are called virtual memory cells. They can produce specific reactions to certain antigens without previous exposure^5,15–19^ and increase NKG2D and granzyme B expression upon IL-12, IL-18, and IL-15 stimulation, performing a bystander killing role in infectious diseases^16,20,21^. In addition, MP CD8^+^ T cells can play an antigen-specific protective role in *Listeria monocytogenes*, Herpes simplex virus (HSV), and *Vaccinia* infections^5,15,18,22^. Additionally, MP CD8^+^ T cells have high affinity for self-antigens, so they can break peripheral tolerance with MP CD4^+^ T cells and develop autoimmune diabetes^23,24^. Overall, previous studies have shown the functions of MP CD8^+^ T cells in disease, but the role of MP CD4^+^ T cells in autoimmune disease has not yet been studied.

Most previous studies have focused on the role of autoantigen-specific T cells in both humans and mice to understand autoimmune diseases and find therapeutic drugs to regulate antigen-specific T cells^25,26^. Interestingly, antigen-nonspecific T cells, including myelin oligodendrocyte glycoprotein (MOG) tetramer–negative CD4^+^ Th17 cells, also infiltrate the central nervous system (CNS) in significant proportions and exacerbate experimental autoimmune encephalomyelitis (EAE) pathogenesis^27–30^. Bystander-activated T cells and Epstein‒Barr virus–specific CD8^+^ T cells are clonally expanded and correlate with disease pathogenesis in the joints of chronic inflammatory arthritis and Sjogren’s syndrome patients^31–33^, raising questions about the role of antigen-nonspecific T cells in autoimmune disease.

In this study, we hypothesized that MP conventional CD4^+^ T cells could be encephalitogenic bystander cells during the development of autoimmune neuroinflammatory disease. First, we examined the heterogeneity of MP CD4^+^ T cells using single-cell RNA sequencing (scRNA-seq) and TCR-sequencing analyses. We found distinct subpopulations of Th1-, Th17-, Treg-, and Tfh-like cells among MP CD4^+^ T cells. These cells responded to IL-1 family cytokines and STAT-activating cytokines, even without TCR stimulation. We further found that CCR6^high^ MP CD4^+^ T cells were the major responders to IL-23 and IL-1β, expressing pathogenic signature genes in a bystander manner and thereby contributing to the development of MOG antigen-specific T-cell-induced EAE. In this context, we identified Bhlhe40, which regulated the production of GM-CSF in CCR6^high^ MP CD4^+^ T cells in a bystander manner to exacerbate EAE pathogenesis. Our findings indicate the pathogenic role of antigen-independent MP CD4^+^ T cells along with antigen-specific T cells during autoimmune neuroinflammatory disease.

## Materials and methods

### Mice

#### Wild type (WT)

C57BL/6J mice were purchased from DBL (Chungcheongbuk-do, Korea), and Rag^–/–^, GM-CSF^–/–^ and 2D2 TCR-transgenic mice were purchased from The Jackson Laboratory (Bar Harbor, ME, USA). CD45.1^+^, Foxp3-GFP mice were provided by Jeehee Youn (Hanyang University). GF and AF mice were purchased from the Microbiome Core Facility of POSTECH (Pohang, Korea). Bhlhe40^–/–^ and Bhlhe40^GFP^ mice were provided by Brian T. Edelson (Washington University). Il1r1^–/–^ mice were provided by Heung-Kyu Lee (KAIST University). Mice were housed and bred in a specific pathogen–free animal facility at Hanyang University under controlled conditions with a constant temperature (21 ± 1 °C) and humidity (50 ± 5%) and a 12-h light/dark cycle with regular chow and autoclaved water. All mouse experimental procedures used in this study were approved by the Institutional Animal Care and Use Committee of Hanyang University (2020-0018A, 2021-005A, 2021-0158A).

### MP CD4^+^ T cell isolation and in vitro activation

MP (TCRβ^+^CD4^+^CD1d tetramer^–^CD25^–^CD62L^low^CD44^high^) CD4^+^ T cells from the spleens of 8- to 12-week-old mice were isolated using a FACS Aria II and FACS Aria Fusion cell sorter (BD Biosciences, Franklin Lakes, NJ, USA). FACS-sorted MP CD4^+^ T cells were stimulated with IL-1β (20 ng/mL, R&D Systems, Minneapolis, MN, USA), IL-23 (20 ng/mL, R&D Systems), IL-12 (20 ng/mL, PeproTech, Rocky Hill, NJ, USA), IL-18 (20 ng/mL, R&D Systems), IL-33 (20 ng/mL, R&D Systems), IL-25 (20 ng/mL, R&D Systems), IL-7 (10 ng/mL, PeproTech, Rocky Hill, NJ, USA), or plate-bound anti-CD3/anti-CD28 (2 μg/mL, BD Biosciences) for 5 days at 37 °C in an incubator.

### Active EAE and adoptive transfer EAE

In the active EAE model, FACS-sorted MP CD4^+^ T cells (γδTCR^–^NK1.1^–^V_β_11^–^TCRβ^+^CD4^+^CD1d tetramer^–^Foxp3^–^CD62L^low^CD44^high^, 5 × 10^5^) from Foxp3-GFP mice were adoptively transferred into 5-week-old female C57BL/6 mice. After the transfer, the mice were immunized with 200 μg of MOG_35-55_ peptide in complete Freund’s adjuvant (Chondrex, Inc., Woodinville, WA, USA). At 0 and 48 h after immunization, the mice were intraperitoneally treated with 500 ng of pertussis toxin (List Biological Laboratories, Inc., Campbell, CA, USA). The animals were scored daily for clinical disease.

In another EAE model established by adoptive transfer, naive (CD4^+^V_β_11^+^CD25^–^CD62L^high^CD44^low^) CD45.1^−^ T cells (1–5 × 10^4^) from 2D2 TCR-transgenic mice were transferred into Rag^–/–^ mice with or without WT CCR6^high^, WT CCR6^low^, Bhlhe40^–/–^ CCR6^high^, GM-CSF^–/–^ CCR6^high^, GM-CSF^–/–^ CCR6^low^, Il1r1^–/–^ CCR6^high^ or Il1r1^–/–^ CCR6^low^ MP CD4^+^ T cells (CD45.1^+^γδTCR^–^NK1.1^–^V_β_11^–^TCRβ^+^CD4^+^CD1d tetramer^–^CD25^–^CD62L^low^ CD44^high^, 1.0 × 10^5^). Before the transfer, CCR6^high^ or CCR6^low^ MP CD4^+^ T cells were primed in vitro with IL-7 (10 ng/mL) and IL-1β (20 ng/mL) for 5–7 days. After the transfer, the mice were immunized with 100 μg of MOG_35−55_ peptide in complete Freund’s adjuvant. At 0 and 48 h after immunization, the mice were intraperitoneally injected with 200 ng of pertussis toxin (List Biological Laboratories, Inc., Campbell, CA, USA). The animals were scored daily for clinical disease as follows^34^: partially limp tail, 0.5; completely limp tail, 1; limp tail and waddling gait, 1.5; paralysis of one hind limb, 2; paralysis of one hind limb and partial paralysis of the other hind limb, 2.5; paralysis of both hind limbs, 3; ascending paralysis, 3.5; paralysis of trunk, 4; moribund, 4.5; and dead, 5. On Day 12 or 13, the mice were sacrificed and perfused with phosphate-buffered saline (PBS). To isolate lymphocytes, the spinal cord and brain were digested with 1 mg/mL collagenase D (11 088 866 001; Sigma‒Aldrich) and DNase I (10 104 159 001; Sigma‒Aldrich) and incubated at 80 RPM on a shaker for 35 min. After enzymatic digestion, lymphocytes were isolated by Percoll (GE Healthcare, Little Chalfont, UK) density gradient centrifugation.

### In vitro MOG reactivity assay (CFSE)

To assess antigen-specific proliferation, 2D2 naïve T cells (CD4^+^V_β_11^+^CD25^–^CD62L^high^CD44^low^), CCR6^high^ or CCR6^low^ MP CD4^+^ T cells (γδTCR^–^NK1.1^–^V_β_11^–^TCRβ^+^CD4^+^CD1d tetramer^–^CD25^–^CD62L^low^ CD44^high^) and CD11c^+^ dendritic cells (MHC-II^+^CD11c^+^) (APCs) from the spleens of 2D2 TCR-transgenic and C57BL/6 mice were isolated using a FACS Aria Fusion cell sorter. Before coculture, naïve and MP CD4^+^ T cells were stained with 1.25 μM carboxyfluorescein succinimidyl ester (CFSE) (Invitrogen, Carlsbad, CA) for 7 min at room temperature. After the incubation, 10% fetal bovine serum (FBS) was added, and the cells were incubated on ice for 3 min. 2D2 naïve T cells and WT CCR6^high^ or WT CCR6^low^ MP CD4^+^ T cells (1 × 10^5^ cells/well) were then washed with PBS and cocultured with CD11c^+^ dendritic cells (5 × 10^4^) with or without 50 μg/mL MOG_35-55_ peptide for 72 h.

### Flow cytometry

Cell-surface staining was performed using the following monoclonal antibodies: anti-CD4 (RM4-5; eBioscience, San Diego, CA, USA, dilution 1:500), anti-CD25 (PC61.5; eBioscience, dilution 1:500), anti-CD44 (IM7; BioLegend, San Diego, CA, USA, dilution 1:500), anti-CD62L (MEL-14; BioLegend, dilution 1:500), anti-CD45 (30-F11, BioLegend, dilution 1:500), anti-CD45.1 (A20; eBioscience, dilution 1:500), anti-TCRβ (H57-597; eBioscience, dilution 1:500), anti-CCR6 (29-2L17; BD, dilution 1:100), anti-CXCR3 (CXCR3-173; BD, dilution 1:100), anti-V_α_3.2 (RR3-16; BioLegend, dilution 1:500), and anti-V_β_11 (RR3-15; BioLegend, dilution 1:500); a PE-conjugated CD1d tetramer (PBS57; NIH, dilution 1:1000) was also used for cell-surface staining. Biotinylated PBS57-loaded and unloaded CD1d monomers were provided by the US National Institutes of Health Tetramer Core facility. For intracellular staining, cells were stimulated with a cell stimulation cocktail (00-4975-03; eBioscience) for 4 h at 37 °C, and then surface markers were stained. Then, the cells were fixed and permeabilized in Cytofix/Cytoperm (554714; BD Bioscience) or a FOXP3/Transcription factor staining buffer set (00-5523-00; eBioscience) for 30 min at 4 °C or RT. Intracellular staining was performed using the following monoclonal antibodies: anti-IL-17A (eBio17B7; eBioscience, dilution 1:200), anti-IFN-γ (XMG1.2; eBioscience, dilution 1:400), anti-GM-CSF (MP1-22E9; BD Biosciences, dilution 1:200), anti-Ki67 (SolA15; eBioscience, dilution 1:500), anti-IL-4 (11B11; BioLegend, dilution 1:200), anti-IL-13 (eBio13A; eBioscience, dilution 1:200), anti-TNF-α (MP6-XT22; BioLegend, dilution 1:500), anti-IL-5 (TRFK5; BioLegend, dilution 1:200), anti-IL-1R1 (35F5; BD Biosciences, dilution 1:100), anti-RORγt (Q31-378; BD Biosciences, dilution 1:100), anti-T-bet (4B10; BioLegend, dilution 1:50), anti-GATA3 (L50-823; BD Bioscience, dilution 3 μL per well) and IgG1 (R2-34; BD Biosciences). Stained cells were analyzed by flow cytometry (FACS Canto II, BD Bioscience), and data were analyzed using FlowJo software version 10.8.0 (TreeStar, Ashland, OR, USA).

### Cytokine measurement (ELISA)

Samples were evaluated using IL-17A ELISA (432501; BioLegend), IL-13 ELISA (88-7137-88; Thermo Fisher), IL-4 ELISA (431104; BioLegend), IL-5 ELISA (431204; BioLegend), IFN-γ ELISA (430801; BioLegend), TNF-α ELISA (430904; BioLegend), and GM-CSF ELISA (432201; BioLegend) kits according to the manufacturers’ instructions. Briefly, microwell plates (Corning Costar; 9018) were coated with capture antibodies overnight at 4 °C and blocked with ELISA diluent 1× for 1 h at room temperature. Samples and twofold serially diluted standards were incubated at room temperature for 2 h, and then detection antibodies and streptavidin-HRP were added. Then, a 1× TMB solution and a stop solution (2 N H_2_SO_4_) were added. The optical density was analyzed at 450 nm. Between all steps, the plates were washed at least 3 times with wash buffer (1× PBS, 0.05% Tween-20).

### Statistical analysis

All data were analyzed in nonparametric analyses using the Mann‒Whitney test or two-way ANOVA in Prism version 8.0 (GraphPad Software, San Diego, CA). Data are presented as the mean ± S.D. or mean ± S.E.M. For all data, significance was defined as *p* ≤ 0.05. Sample size and statistical information are provided in each figure legend.

## Results

### Single-cell RNA sequencing identifies clearly distinct effector-like subpopulations in steady-state MP conventional CD4^+^ T cells

**Fig. 1:**
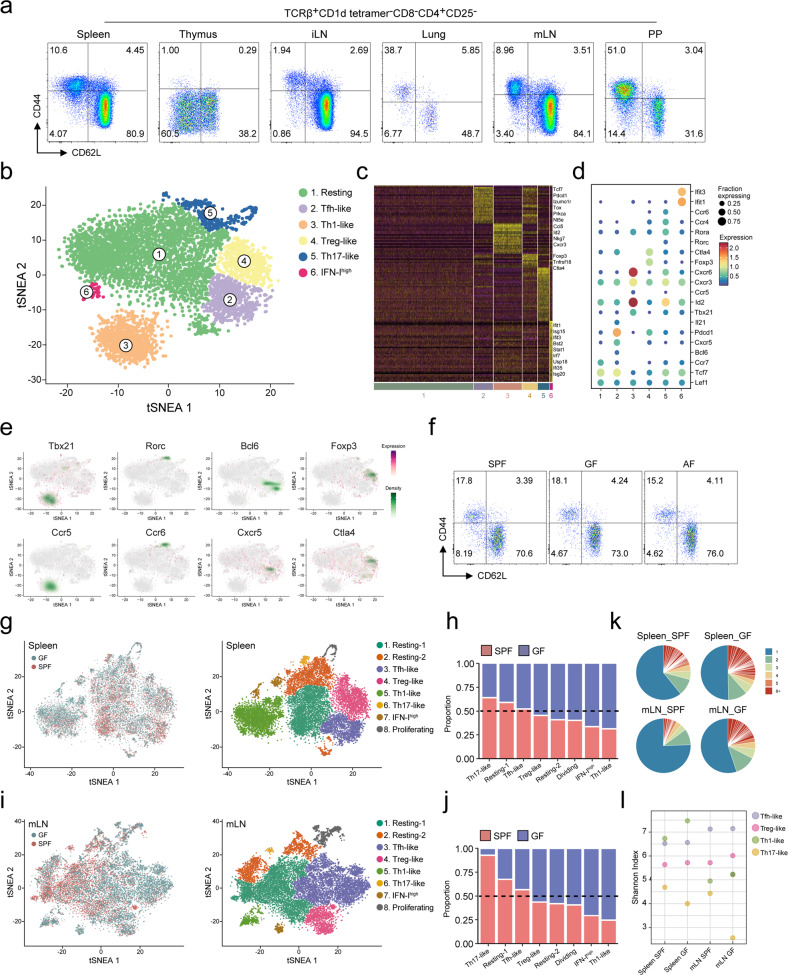
Single-cell RNA sequencing identifies clearly distinct effector-like subpopulations in steady-state MP CD4^+^ T cells. **a** Representative flow cytometry plots showing the population of memory-phenotype (MP) CD4^+^ T cells (TCRβ^+^CD1d tetramer^–^CD8^–^CD4^+^CD25^–^CD62L^low^CD44^high^) in murine spleens, thymuses, inguinal lymph nodes (iLNs), lungs, mesenteric lymph nodes (mLNs), and Peyer’s patches (PP). **b** tSNE plot of splenic MP CD4^+^ T cells isolated from 10-week-old specific-pathogen-free (SPF) mice. **c** Heatmap of differentially expressed transcripts in splenic MP CD4^+^ T cells. **d** Expression of selected genes used to define MP CD4^+^ T cell clusters. **e** Differential expression of transcription factors and chemokine receptors in splenic MP CD4^+^ T cell clusters. **f** Flow cytometry plots of splenic MP CD4^+^ T cells (TCRβ^+^CD1d tetramer^–^CD8^–^CD4^+^CD25^–^CD62L^low^CD44^high^) from SPF, GF, and AF mice. **g** tSNE plot and **h** proportion of integrated splenic MP CD4^+^ T cells isolated from 10-week-old SPF and GF mice. **i** tSNE plot and **j** proportion of integrated mLN MP CD4^+^ T cells isolated from SPF and GF mice. **k** Pie chart representing the clonal size distribution of MP CD4^+^ T cells. **l** Diversity of the TCR repertoire of MP CD4^+^ T cell subsets from SPF and GF mice.

Steady-state, unprimed specific-pathogen-free (SPF)-housed mice had a significant proportion of MP CD4^+^ T cells (TCRβ^+^CD1d tetramer^–^CD8^–^CD4^+^CD25^–^CD44^high^CD62L^low^) in the spleen, thymus, inguinal lymph nodes (iLNs), mesenteric lymph nodes (mLNs), Peyer’s patches (PPs), and lung tissues (Fig. 1a and Supplementary Fig. 1a). The proportion and number of CD4^+^ T cells increased with age in all tissues (Supplementary Fig. 1). To identify and focus on the characteristics of MP CD4^+^ T cells, we used fluorescence-activated cell sorting (FACS) to sort MP CD4^+^ T cells from the spleen of 10-week-old C57BL/6 mice and performed scRNA-seq with paired V(D)J sequencing of the T cell receptor (Supplementary Fig. 2a-d). Additionally, we generated a pipeline to filter out PLZF and TCR V_α_14-J_α_18 (TRAV11-TRAJ18)-expressing cells, a well-known key transcription factor for the development of NKT and MAIT cells^35–37^. An unbiased clustering analysis revealed clearly distinct effector T cell–like subpopulations (Fig. 1b) and differentially expressed genes (DEGs) defining each cluster (Fig. 1c, d). A Gene Ontology (GO)/Kyoto Encyclopedia of Genes and Genomes (KEGG) analysis of each cluster indicated that Clusters 3 and 5 contained Th1- and Th17-like populations, respectively, with significant enrichment in “chemokine-mediated signaling pathway” and “cellular response to interleukin-1” (Supplementary Fig. 3). In addition, lineage-specific transcription factors and chemokine receptors were localized in each cluster (Fig. 1e and Supplementary Fig. 4), suggesting that MP CD4^+^ T cells are composed of Th1-, Th17-, Tfh-, and Treg-like subpopulations. To examine whether the subpopulations of steady-state MP CD4^+^ T cells depended on germ or food antigens, we analyzed the proportion of CD44^high^CD62L^low^ MP CD4^+^ T cells in the tissues of SPF-, GF-, and AF-housed mice using flow cytometry. The proportion of total MP CD4^+^ T cells was almost identical in the spleen and other tissues except the mLNs and PPs (Fig. 1f and Supplementary Fig. 5a, b), indicating that the generation of splenic MP CD4^+^ T cells is not affected by the microbiome or food antigen stimulation. Our scRNA-seq analysis further confirmed that the SPF- and GF-housed mice had identical proportions of distinct effector-like subpopulations of splenic MP CD4^+^ T cells (Fig. 1g, h), but the proportion of Th17-like MP cells in the mLNs of GF-housed mice was much lower than that in the mLNs of SPF-housed mice (Fig. 1i, j). In agreement, the proportion of CCR6^high^ or RORγt^+^ MP CD4^+^ T cells in the mLNs of GF-housed mice was lower than that of SPF-housed mice, suggesting that the generation of gut MP CD4^+^ T cells is specifically dependent on germ exposure (Supplementary Fig. 6). The TCR clonal diversity of MP CD4^+^ T cells in GF-housed mice, especially that of the Th17-like population in the mLNs, was reduced compared with that in SPF-housed mice, although comparable diversity was observed in the spleen (Fig. 1k, l). There seemed to be no significant clonal expansion in MP subpopulations. Together, these data indicate that steady-state splenic MP CD4^+^ T cells contain heterogeneous subpopulations of Th1-, Th17-, Tfh-, and Treg-like cells that express effector molecules and exist independently of the gut microbiome and food antigens.

### Differential innate-like effector functions of MP CD4^+^ T cells after exposure to IL-1 family and STAT-activating cytokines

**Fig. 2:**
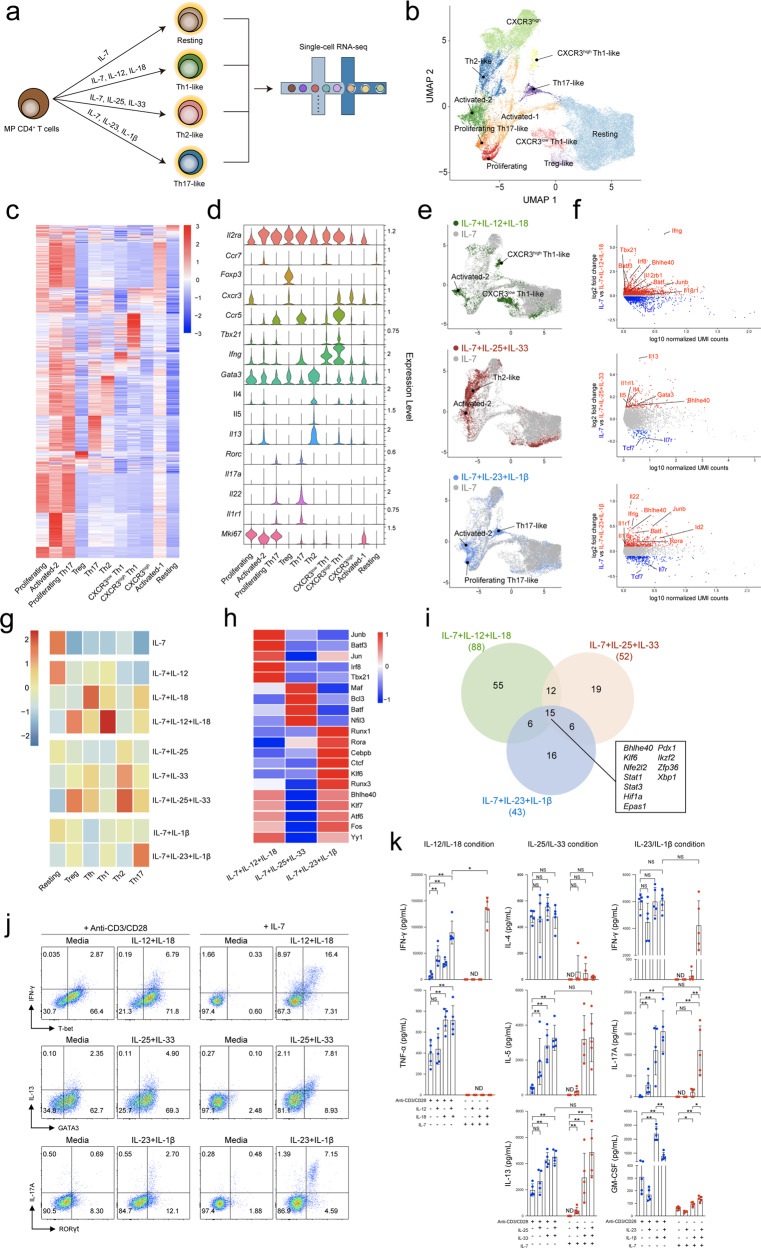
Differential innate-like effector functions of MP CD4^+^ T cells after exposure to IL-1 family and STAT-activating cytokines. **a** Overview of the experimental design. **b** UMAP representation of MP CD4^+^ T cells stimulated with IL-12 and/or IL-18 and IL-25 and/or IL-33 and IL-23 and/or IL-1β in the presence of IL-7. **c** Heatmap and **d** violin plots of differentially expressed transcripts in a cluster. **e** Individual cytokine conditions visualized with UMAP. **f** MA plots of differentially expressed genes comparing IL-7 versus IL-12/18, IL-25/33 or IL-23/1β. **g** Heatmap representing gene expression of resting-, Tfh-, Th1-, Th2-, Th17- and Treg-related gene signatures in each cytokine condition. **h** Heatmap representing transcriptional activity. **i** Venn diagram of transcriptional regulators predicted by IPA. Numbers indicate the number of genes in each gate. **j** MP CD4^+^ T cells (TCRβ^+^CD1d tetramer^–^CD8^–^CD4^+^CD25^–^CD62L^low^CD44^high^) were cultured for 5 days with IL-12 and/or IL-18 and IL-25 and/or IL-33 and IL-23 and/or IL-1β in the presence of IL-7 or anti-CD3/anti-CD28. Representative flow cytometry plots showing the expression of effector lineage markers in each cytokine condition. **k** IFN-γ, TNF-α, IL-4, IL-5, IL-13, IL-17A, and GM-CSF concentrations were measured by ELISA (*n* = 5, 5 independent experiments). Data are presented as the mean ± S.D. *p* Values were calculated using the Mann‒Whitney *U*-test (*p < 0.05, **p < 0.01).

Previous studies have shown that alarmin cytokines, such as IL-12/IL-18, initiate type 1 inflammation, while IL-25/IL-33/TSLP stimulate type 2 inflammation, and IL-23/IL-1β induce type 3 inflammation, activating specific subsets of ILCs or innate immune cells^38,39^. In addition, IL-1 family cytokines and STAT activators induce effector cytokine production in Th1, Th2, and Th17 cells without TCR stimulation^40,41^. To determine the bystander effector functions of MP CD4^+^ T cells in response to specific sets of these cytokines, we performed scRNA-seq of MP CD4^+^ T cells cultured in conditioned medium containing the cytokines IL-12/IL-18, IL-25/IL-33, or IL-23/IL-1β (Fig. 2a and Supplementary Fig. 2e). Additionally, IL-7 was added to the culture medium for T-cell survival and maintenance^42^. A merged uniform manifold approximation and projection (UMAP) plot illustrated the phenotypic characteristics of Th1-, Th2-, Th17-, and Treg-like subpopulations (Fig. 2b, c), which expressed lineage-specific genes (Fig. 2d). Each cytokine set induced distinct subpopulations localized exclusively in the plot (Fig. 2e). We further confirmed the expression of selected gene sets related to the Th1, Th2, and Th17 lineages in each bystander-activated condition, which showed that type 1, 2, and 3 cytokines could upregulate lineage-specific genes in MP CD4^+^ T cells (Fig. 2f, g). A single-cell regulatory network inference and clustering (SCENIC) analysis of each cytokine condition revealed a significant level of common or specific transcription factor activity (Fig. 2h). In addition, an Ingenuity pathway analysis (IPA) identified the predicted transcriptional regulators in each condition and speculated that the target transcription factors included Bhlhe40, which could be a potent regulator of bystander activation in MP CD4^+^ T cells (Fig. 2i). To evaluate the effector functions of MP CD4^+^ T cells, we treated MP CD4^+^ T cells with the cytokine sets and/or anti-CD3/anti-CD28 antibodies (Fig. 2j, k). Consistently, MP CD4^+^ T cells responded to IL-12/IL-18, IL-25/IL-33, and IL-23/IL-1β without TCR stimulation, and the levels of T-bet^+^IFN-γ^+^, GATA3^+^IL-13^+^, and RORγt^+^IL-17^+^ cells were increased in these conditions compared to TCR-stimulated conditions (Fig. 2j). However, TNF-α was produced only in the presence of TCR stimulation and IFN-γ in culture supernatant from IL-12/IL-18-conditioned MP CD4^+^ T cells, and IL-4 was also significantly detected with TCR stimulation but not in the bystander condition with IL-25/IL-33. Similarly, granulocyte-macrophage colony-stimulating factor (GM-CSF) was secreted more efficiently with IL-23/IL-1β and TCR stimulation than with the cytokines alone (Fig. 2k). Collectively, these results indicate that steady-state MP CD4^+^ T cells have functional heterogeneity, producing innate-like responses to various sets of IL-1 family and STAT-activating cytokines even in the absence of T cell receptor stimulation, and suggest possible potent transcriptional regulators that control MP CD4^+^ T cell effector functions.

### Potential responder MP CD4^+^ T cells express distinct chemokine receptors upon IL-12/IL-18 and IL-23/IL-1β stimulation

**Fig. 3:**
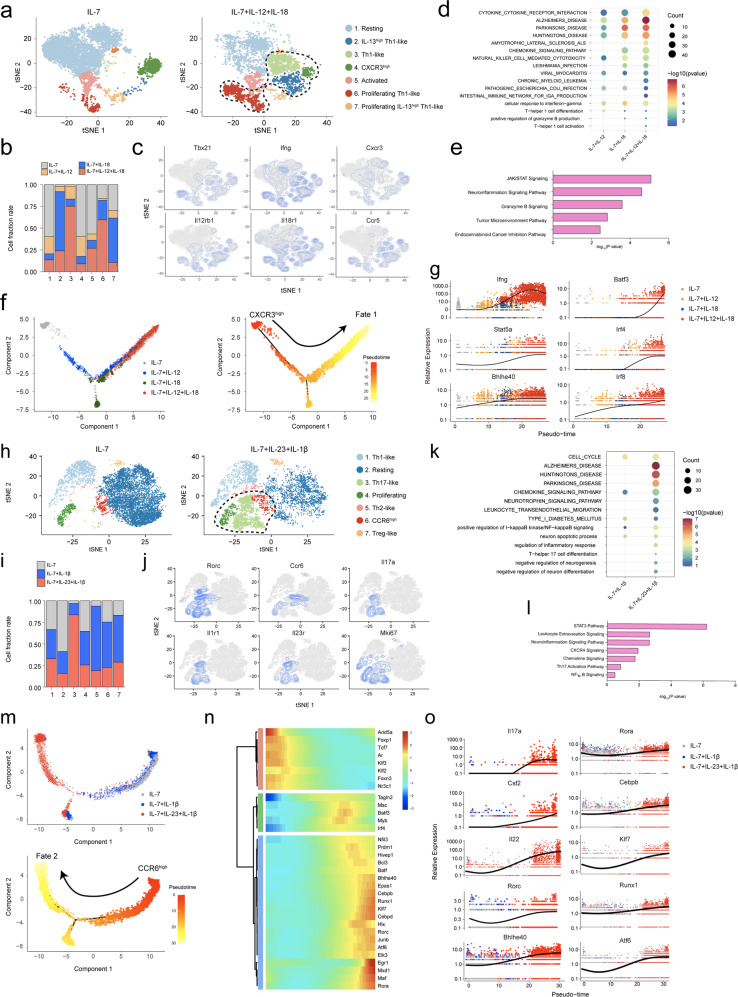
Potential responder MP CD4^+^ T cells express distinct chemokine receptors upon IL-12/IL-18 and IL-23/IL-1β stimulation. **a** tSNE plots and **b** proportion of MP CD4^+^ T cells stimulated with IL-12 and IL-18 in the presence of IL-7 for 5 days. **c** Expression of selected Th1-related genes. **d** Selected KEGG/GO terms in Clusters 2, 3, 4 and 6 and **e** Ingenuity pathway analysis (IPA) of Clusters 3, 4 and 6 of IL-12- and IL-18-responsive MP CD4^+^ T cells. **f** Pseudotime trajectory: each cell is colored to indicate its pseudotime value and **g** the expression level of the related genes. **h** tSNE plots and **i** proportion of MP CD4^+^ T cells stimulated with IL-23 and IL-1β in the presence of IL-7 for 5 days. **j** Expression of selected Th17-related genes. **k** Selected KEGG/GO terms and **l** IPA of IL-23- and IL-1β-stimulated MP CD4^+^ T cells (Clusters 3, 4, and 6). **m** Pseudotime trajectory: each cell is colored to indicate its pseudotime value, **n** transcription factor activity and **o** expression level of related genes.

To determine which MP CD4^+^ T cells are potential responders to IL-1 family and STAT-activating cytokines, we analyzed the subpopulations of expanding or responding clusters and performed trajectory analyses. Control MP CD4^+^ T cells cultured with IL-7 produced a significantly separate population of CXCR3^high^ cells. In IL-12/IL-18-conditioned MP CD4^+^ T cells, the CXCR3^high^ cell population was reduced, and the IFN-γ-expressing or IL-13^high^ Th1 and proliferating Th1 populations were greatly increased (Fig. 3a, b). The Th1 signature genes *Tbx21, Ifng, Cxcr3, Il2rb1, Il18r1*, and *CCR5* were highly expressed by IL-12/IL-18-conditioned MP CD4^+^ T cells (Fig. 3c). A GO/KEGG analysis indicated that the related gene sets in these cells were enriched including “Cellular response to interferon-gamma”, “Cytokine cytokine receptor interaction”, “Alzheimer’s disease”, and “Parkinson’s disease” (Fig. 3d). An IPA returned terminologies related to cytokines and inflammation, such as “JAK/STAT signaling” and “neuroinflammation signaling pathway”, and to cytotoxic response, including “Granzyme B signaling” (Fig. 3e). In a pseudotime trajectory analysis, MP CD4^+^ T cells formed a continuous progression that started in CXCR3^high^ cells and gradually progressed toward Fate 1, which expressed *Ifng, Stat5a, Bhlhe40, Batf3, Irf4* and *Irf8* (Fig. 3f, g). Similarly, a CCR6^high^ cluster was present in IL-7-conditioned MP CD4^+^ T cells, and the level of its Th17-like cluster was specifically increased by IL-23 and IL-1β (Fig. 3h, i). The Th17 signature genes *Rorc, Ccr6, Il17a Il1r1, and Il23r* and the proliferation marker *Mki67* were expressed by these cells (Fig. 3j). A GO/KEGG analysis predicted that the related gene sets in IL-23/IL-1β-cultured MP CD4^+^ T cells had enrichment of the terms “Alzheimer’s disease,” “Parkinson’s disease,” and “Huntington’s disease”, which are neurological diseases, and enrichment of “cytokine signaling pathway” (Fig. 3k). An IPA more clearly identified the related pathways, including “STAT3 pathway,” “leukocyte extravasation signaling,” “neuroinflammation signaling pathway,” “chemokine signaling,” and “Th17 activation pathway” (Fig. 3l). In a trajectory analysis, CCR6^high^ cells seemed to be the starting point, and then the cells gradually differentiated toward Fate 2 (Fig. 3m), which expressed pathogenic Th17-related genes such as *Rorc, Il17a, Csf2, Il22, Bhlhe40, Rora and Cebpb* with increased activities (Fig. 3n) and expression levels (Fig. 3o) in related transcriptomes. These results collectively reveal that Th1-like and Th17-like MP CD4^+^ T cells expressing different chemokine receptors and respond specifically to IL-12/IL-18 and IL-23/IL-1β by exerting effector functions.

### Steady-state CCR6^high^ memory-phenotype CD4^+^ T cells undergo bystander activation by IL-23 and IL-1β to become pathogenic Th17-like cells

**Fig. 4:**
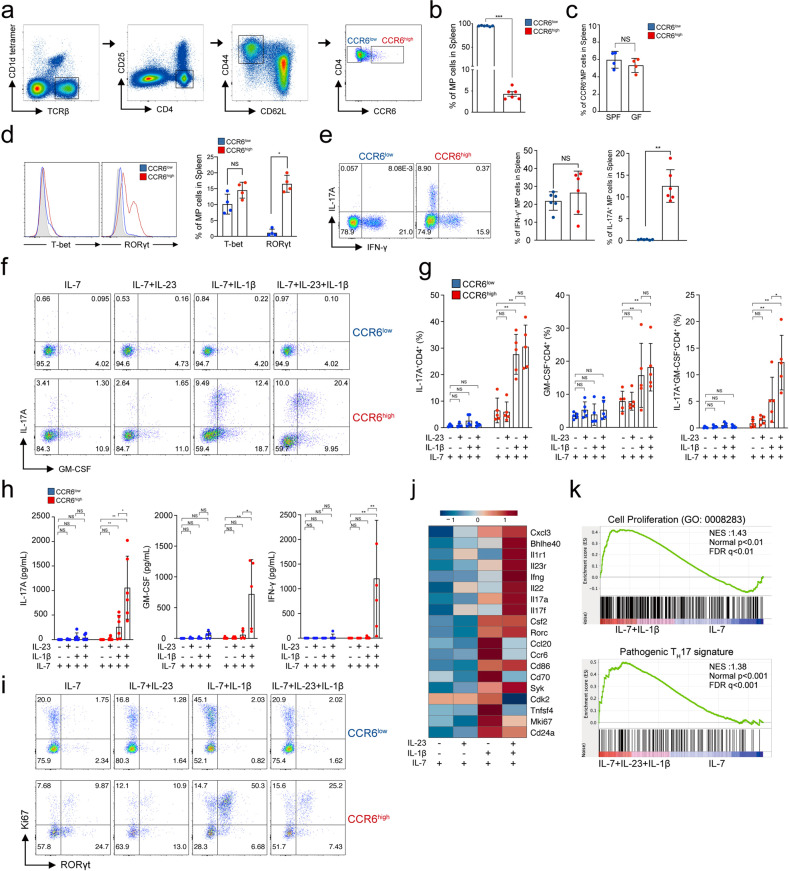
Steady-state CCR6^high^ MP CD4^+^ T cells undergo bystander activation by IL-23 and IL-1β to become pathogenic Th17-like cells. **a** Gating strategy for CCR6^high^ and CCR6^low^ MP CD4^+^ T cells from SPF mice. **b** The percentage of CCR6-expressing cells in steady-state splenic MP CD4^+^ T cells (*n* = 6). **c** The expression of CCR6 in SPF mouse- vs. GF mouse-derived MP CD4^+^ T cells (*n* = 4). **d** Expression levels of the transcription factors T-bet and RORγt in FACS-sorted CCR6^high^ and CCR6^low^ MP CD4^+^ T cells and **e** cytokine expression and the average proportion of CCR6^high^ MP CD4^+^ T cells vs. CCR6^low^ MP CD4^+^ T cells (*n* = 6). CCR6^high^ and CCR6^low^ MP CD4^+^ T cells were stimulated with IL-23 and/or IL-1β in the presence of IL-7 for 5 days. **f** The representative proportion and **g** the average value of cytokine-producing cells (*n* = 5). **h** The concentrations of IL-17A, GM-CSF, and IFN-γ were analyzed by ELISA (*n* = 5). **i** The proportions of RORγt^+^ and Ki67^+^ cells in CCR6^high^ and CCR6^low^ MP CD4^+^ T cells. **j** Heatmap of selected genes. **k** Gene set enrichment analysis (GSEA) pathway enrichment plot related to “Cell Proliferation” and “Pathogenic T_H_17 signature” by bulk RNA-seq analysis. *q* false discovery rate, NES normalized enrichment score. Data are presented as the mean ± S.D. All *p* values were calculated using the Mann‒Whitney *U*-test (ND not detected, NS not significant; *p < 0.05, **p < 0.01, ***p < 0.001).

Since scRNA-seq predicted that CCR6^high^ cells were the major cells responding to IL-23 and IL-1β, we sorted splenic CCR6^high^ and CCR6^low^ MP CD4^+^ T cells (Fig. 4a, b) and then determined their proportions in steady-state SPF- and GF-housed mice (Fig. 4c). We found that splenic CCR6^high^ MP CD4^+^ T cells were independent of the gut. CCR6^high^ MP CD4^+^ T cells showed greater expression of RORγt and the cytokine receptors for IL-23 and IL-1β than CCR6^low^ cells and had comparable expression of T-bet (Fig. 4d and Supplementary Fig. 7). Steady-state CCR6^high^ MP CD4^+^ T cells, but not CCR6^low^ cells, expressed IL-17A (Fig. 4e), suggesting that CCR6^high^ MP CD4^+^ T cells are Th17-like cells. Further stimulation by IL-23 and IL-1β induced IL-17A and GM-CSF expression (Fig. 4f, g), confirming that CCR6^high^ MP CD4^+^ T cells produce pathogenic cytokines in a bystander manner. The amount of cytokine secreted, as determined by ELISA, consistently showed that CCR6^high^ MP CD4^+^ T cells but not CCR6^low^ cells significantly produced IL-17A, GM-CSF, and IFN-γ and that IL-23 and IL-1β induced synergistic effect which are important for the pathogenicity of the disease by regulating the expression levels of IL1R1 and IL23R (Fig. 4h and Supplementary Fig. 7). In addition, IL-1β greatly enhanced RORγt expression in CCR6^high^ MP CD4^+^ T cells but not CCR6^low^ cells, whereas IL-23 somewhat inhibited the proportion of Ki67-expressing cells, suggesting that IL-1β is important in the proliferation of CCR6^high^ MP CD4^+^ T cells (Fig. 4i). To further confirm the functions of IL-23 and IL-1β in MP CD4^+^ T cells, we performed bulk RNA-seq with bystander-activated MP CD4^+^ T cells exposed to IL-23 and IL-1β. As shown by a heatmap DEG analysis, IL-1β increased the expression of proliferation-related genes such as *Mki67* and *cdk2*, whereas IL-23 alone did not have any significant effects on gene expression (Fig. 4j). IL-23 and IL-1β together significantly induced the expression of pathogenic genes such as *Bhlhe40, Il1r1, Csf2*, *Ifng, Il22* and *Il17a*. In support of this finding, a gene set enrichment analysis (GSEA) pathway enrichment plot of IL-1β vs. IL-23 and IL-1β demonstrated that the enrichment score for cell proliferation was higher with IL-1β and that the score for the pathogenic Th17 signature with IL-23 and IL-1β was higher than that with control treatment (Fig. 4k). Through these results, we determined that steady-state CCR6^high^ MP CD4^+^ T cells, which show Th17-like characteristics, are the major bystander-activated cells responding to IL-23 and IL-1β, which potentiate the cells’ pathogenic character.

### CCR6^high^ MP CD4^+^ T cells exacerbate autoimmune neuroinflammation in a bystander manner

**Fig. 5:**
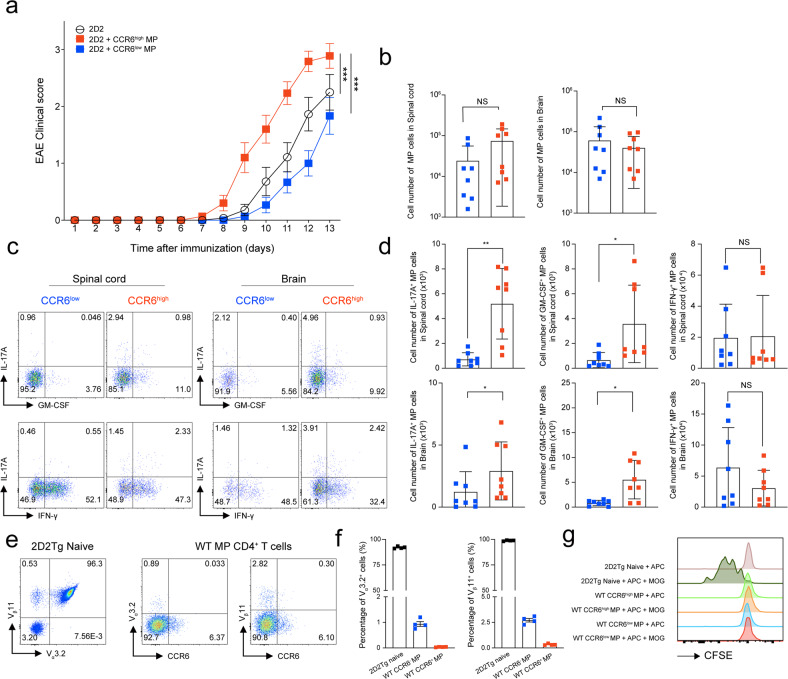
CCR6 ^high^ MP CD4^+^ T cells exacerbate autoimmune neuroinflammation in a bystander manner. **a** Naïve CD4^+^ T cells (5 × 10^4^) from 2D2 transgenic mice were adoptively transferred, with or without CCR6^high^ or CCR6^low^ MP CD4^+^ T cells (1 × 10^5^, CD45.1^+^γδTCR^–^NK1.1^–^V_β_11^–^TCRβ^+^CD4^+^CD1d tetramer^–^CD25^–^CD62L^low^CD44^high^), into Rag^–/–^ mice that were immunized with MOG_35-55_ in CFA. The EAE clinical score was monitored daily (*n* = 15). **b** Absolute cell numbers of infiltrated CD45.1^+^MP CD4^+^ T cells in the spinal cord and brain (*n* = 8). **c** Representative plots and **d** absolute cell numbers of IL-17A-, GM-CSF-, and IFN-γ^–^producing cells in MP CD4^+^ T cells isolated from the spinal cord and brain on Days 12–13 after immunization (*n* = 8). **e** Representative dot plots and **f** average value showing the percentage of 2D2 TCR (V_α_3.2^+^ and V_β_11^+^) in spleens from 2D2 transgenic mice and C57BL/6 wild-type mice (*n* = 4). **g** FACS-sorted 2D2 naïve CD4^+^ T cells and CCR6^high^ or CCR6^low^ MP CD4^+^ T cells were cultured with CD11c^+^ dendritic cells (MHC-II^+^CD11c^+^) with or without the MOG_35-55_ peptide (50 μg/ml) for 3 days. CFSE levels were measured by flow cytometry. Data are presented as the mean ± S.E. in a and the mean ± S.D. in **b**, **d**, **f**. *p* Values were calculated using two-way ANOVA or the Mann‒Whitney *U*-test (NS not significant; *p < 0.05, **p < 0.01, ***p < 0.001).

To reveal the importance of splenic MP CD4^+^ T cells during an autoimmune disease, we first induced active EAE in 5-week-old mice, which have a lower proportion of MP CD4^+^ T cells than 10-week-old mice. In this mouse model, we compared the disease severity of control mice with that of mice that received an additional adoptive transfer of Treg-deleted (Foxp3^–^) MP CD4^+^ T cells from 10-week-old Foxp3-GFP mice. EAE was rapidly induced and progressed following the transfer of the additional MP CD4^+^ T cells from 10-week-old mice, suggesting that MP CD4^+^ T cells could be an important contributor to MOG_35-55_-induced EAE pathogenesis (Supplementary Fig. 8a). In support of this hypothesis, Rag^–/–^ mice adoptively transferred with MOG-TCR transgenic (2D2) naïve CD45.1^–^V_β_11^+^CD4^+^ T cells and Treg-deleted MP CD4^+^ T cells (CD45.1^+^CD4^+^) showed a more severe EAE phenotype than mice that received only 2D2 naïve T cells (Supplementary Fig. 8b), suggesting that MP CD4^+^ T cells contribute to the pathogenesis of EAE. Based on our previous results, we hypothesized that CCR6^high^ MP CD4^+^ T cells are the major pathogenic subpopulation contributing to EAE disease progression. To test this hypothesis, we transferred MOG antigen-nonspecific CCR6^high^ or CCR6^low^ MP CD4^+^ T cells (CD45.1^+^CD4^+^, gating strategy and purity are shown in Supplementary Fig. 9) and 2D2 naïve CD4^+^ T cells into Rag^–/–^ mice. The additional transfer of CCR6^high^ MP CD4^+^ T cells exacerbated EAE development compared with 2D2 transfer alone or the additional transfer of CCR6^low^ MP CD4^+^ T cells (Fig. 5a). Interestingly, the number of transferred MP CD4^+^ T cells that appeared in the spinal cord and brain tissue did not differ between conditions (Fig. 5b). However, CCR6^high^ MP CD4^+^ T cells produced significantly more cytokines, particularly IL-17A and GM-CSF, in the spinal cord and brain tissue than CCR6^low^ MP CD4^+^ T cells (Fig. 5c, d). In addition, CCR6^high^ MP CD4^+^ T cells alone could not induce EAE with MOG immunization (Supplementary Fig. 10). Therefore, CCR6^high^ MP CD4^+^ T cells, along with antigen-specific T cells, contribute to the pathogenicity of autoimmune neuroinflammation by expressing pathogenic cytokines such as IL-17A and GM-CSF. To clarify the antigen-independent activation of CCR6^high^ MP CD4^+^ T cells in an EAE mouse model, we examined the expression level of the 2D2 TCR (V_α_3.2^+^ and V_β_11^+)^, which is predominantly expressed by 2D2 naïve CD4^+^ T cells. Indeed, CCR6^high^ MP CD4^+^ T cells barely expressed V_α_3.2^+^ and V_β_11^+^ (Fig. 5e, f). In agreement with this result, we confirmed that steady-state CCR6^high^ and CCR6^low^ MP CD4^+^ T cells did not respond to the MOG_35-55_ antigen (Fig. 5g). Collectively, these results suggest that CCR6^high^ MP CD4^+^ T cells infiltrate CNS tissue and exacerbate autoimmune neuroinflammation in a bystander manner.

### Innate-like effector functions of CCR6^high^ MP CD4^+^ T cells are conferred by the Bhlhe40/GM-CSF axis

**Fig. 6:**
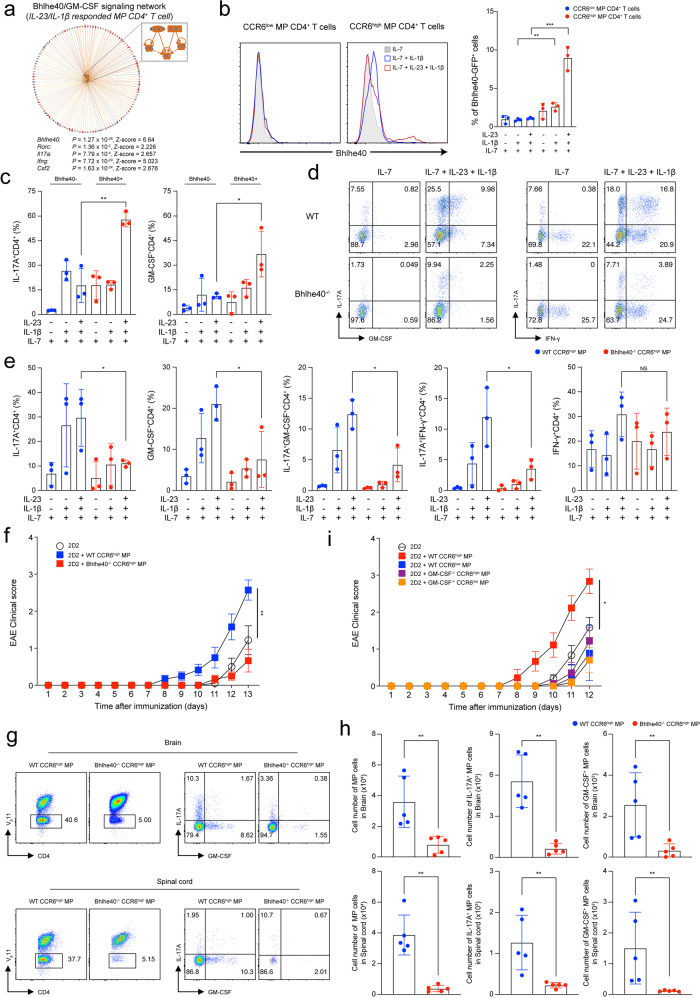
Innate-like effector functions of CCR6^high^ MP CD4^+^ T cells are conferred by the Bhlhe40/GM-CSF axis. **a** Predicted upstream network of IL-23- and IL-1β-responsive MP CD4^+^ T cells by IPA. **b** Representative histogram and average value showing the percentage of Bhlhe40-positive in CCR6^high^ and CCR6^low^ MP CD4^+^ T cells induced by IL-23/IL-1β stimulation without TCR engagement (*n* = 3). **c** Representative percentages of IL-17A- and GM-CSF-expressing cells in Bhlhe40^GFP^-positive and Bhlhe40^GFP^-negative cells in CCR6^high^ MP CD4^+^ T cells induced by IL-23/IL-1β without TCR engagement for 5 days (*n* = 3). **d** Representative flow cytometry plots showing the cytokine expression and **e** average proportion of WT CCR6^high^ MP CD4^+^ T cells vs. Bhlhe40^–/–^ CCR6^high^ MP CD4^+^ T cells (*n* = 3). **f** Naïve CD4^+^ T cells (5 × 10^4^) from 2D2 transgenic mice were adoptively transferred, with or without WT CCR6^high^ or Bhlhe40^–/–^ CCR6^high^ MP CD4^+^ T cells (1 × 10^5^, CD45.1^+^γδTCR^–^NK1.1^–^V_β_11^–^TCRβ^+^CD4^+^CD1d tetramer^–^CD25^–^CD62L^low^CD44^high^), into Rag^–/–^ mice that were immunized with MOG_35-55_ in CFA. The EAE clinical score was monitored daily (*n* = 5). **g** Representative flow cytometry plots and **h** absolute cell numbers of infiltrated 2D2 TCR (V_β_11^+^)^–^ MP CD4^+^ T cells (gating from CD45^+^CD4^+^) and IL-17A-, GM-CSF-, and IFN-γ^–^producing cells in the spinal cord and brain on Day 13 after immunization (*n* = 5). **i** Naïve CD4^+^ T cells (5 × 10^4^) from 2D2 transgenic mice were adoptively transferred, with or without WT CCR6^high^, WT CCR6^low^, GM-CSF^–/–^ CCR6^high^ or GM-CSF^–/–^ CCR6^low^ MP CD4^+^ T cells (1 × 10^5^, CD45.1^+^γδTCR^–^NK1.1^–^V_β_11^–^TCRβ^+^CD4^+^CD1d tetramer^–^CD25^–^CD62L^low^CD44^high^), into Rag^–/–^ mice that were immunized with MOG_35-55_ in CFA. The EAE clinical score was monitored daily (n = 9). Data are presented as the mean ± S.E.M. in F and I and the mean ± S.D. in **b**–**e**, **g**, **h**. Values were calculated using two-way ANOVA or the Mann‒Whitney *U*-test (NS not significant; *p < 0.05, **p < 0.01, ***p < 0.001).

Among the candidate genes involved in the bystander activation of CCR6^high^ MP CD4^+^ T cells, the Bhlhe40/GM-CSF axis was identified to potentially give rise to the pathogenic function of CCR6^high^ MP CD4^+^ T cells induced by IL-23 and IL-1β without TCR stimulation (Fig. 6a). Bhlhe40 has been reported to be an important transcription factor for the production of pathogenic cytokines, such as IL-17 and GM-CSF, and EAE^29,43–45^. We hypothesized that Bhlhe40 could mediate the pathogenic function of CCR6^high^ MP CD4^+^ T cells induced by IL-23 and IL-1β. By using Bhlhe40^GFP^ mice, we found that CCR6^high^ MP CD4^+^ T cells activated by IL-23 and IL-1β showed a significantly increased level of Bhlhe40 (Fig. 6b). Interestingly, Bhlhe40^GFP^ positive T cells primarily produced effector cytokines, including IL-17A and GM-CSF, compared to Bhlhe40^GFP^-negative T cells (Fig. 6c). Similarly, Bhlhe40^–/–^ CCR6^high^ MP CD4^+^ T cells showed markedly reduced IL-17A and GM-CSF production compared to WT cells (Fig. 6d, e), suggesting that Bhlhe40 is an important transcriptional regulator of the pathogenic functions of bystander CCR6^high^ MP CD4^+^ T cells. To confirm the in vivo relevance, we transferred WT CCR6^high^ or Bhlhe40^–/–^ CCR6^high^ MP CD4^+^ T cells along with 2D2 naïve CD4^+^ T cells into Rag^–/–^ mice. Bhlhe40^–/–^ CCR6^high^ MP CD4^+^ T cells showed abrogated functions in exacerbating EAE disease compared with WT CCR6^high^ MP CD4^+^ T cells (Fig. 6f). Interestingly, the number of Bhlhe40^–/–^ CCR6^high^ MP CD4^+^ T cells expressing IL-17A and GM-CSF in the spinal cord and brain tissue was significantly reduced compared to that of WT CCR6^high^ MP CD4^+^ T cells (Fig. 6g, h). Consistent with previous reports noting that Bhlhe40 regulates GM-CSF production with a positive correlation with the *Csf2 gene* locus^29,43,46^, transferred GM-CSF-deficient CCR6^high^ MP CD4^+^ T cells showed a decreased contribution to EAE pathogenesis (Fig. 6i). Collectively, these results suggest that Bhlhe40 drives the production of GM-CSF in CCR6^high^ MP CD4^+^ T cells, endowing them with innate-like pathogenic functions.

## Discussion

In this study, we intensively validated the characteristics of steady-state MP CD4^+^ T cells using scRNA-seq to unveil their innate-like effector functions in autoimmune disease. We found clearly distinct effector-like subpopulations in steady-state splenic MP CD4^+^ T cells that were independent of the microbiome and food antigens. MP CD4^+^ T cells can undergo bystander activation by different sets of IL-1 family and STAT-activating cytokines. Specific chemokine receptor expressing cells are defined as potential responder cells to each set of cytokines, and we focused on CCR6^high^ MP CD4^+^ T cells to further validate their functions in responding to IL-23/IL-1β. We demonstrated that steady-state CCR6^high^ MP CD4^+^ T cells had innate-like effector functions that exacerbated EAE disease progression in a bystander manner, in the presence of antigen-specific T cells. We suggest that Bhlhe40 is a pivotal transcriptional regulator that governs GM-CSF production in bystander-activated CCR6^high^ MP CD4^+^ T cells, exacerbating EAE development. Overall, our results reveal the innate lymphoid cell-like immunological functions of steady-state MP CD4^+^ T cells in autoimmune disease.

Innate T cells such as natural killer T (NKT), mucosal-associated invariant T (MAIT), and γδ T cells have limited TCR gene usage compared to conventional T cells, recognizing complexes of nonpeptide antigens such as glycolipids, phospho-antigens, and vitamin B metabolites, respectively^47–49^. These innate T cells are derived from the thymus and can induce robust cytokine production. Previously, innate lymphocytes, such as NKT17 cell, γδT17 cell, and ILC3 subsets, were defined to commonly express IL-17 and RORγt^50–52^. Furthermore, a recent study reported a novel subset of αβ-γδ coexpressing T cells that recognize MHC-restricted peptide antigens and produce the effector cytokines IL-17A, GM-CSF, and IFN-γ in response to IL-23 and IL-1β stimulation^53^. Additionally, another group found that natural Th17 cells and γδ T cells expanded after candidiasis infection in the oral cavity^54^. In this context, we carefully eliminated the possibility of innate T cell contamination by sorting MP conventional CD4^+^ T cells using NKT cell (CD1d tetramer), γδ T cell, αβ-γδ T cell, ILC, and MAIT cell (TCRβ^+^CD8^–^CD4^+^CD25^–^CD44^high^CD62L^low^) exclusion gates. We confirmed that PLZF/CD1d tetramer negative steady-state MP CD4^+^ T cells still existed as a heterogeneous population containing CCR6^high^RORγt^+^IL-17A^+^ cells, which primarily undergo bystander activation by IL-23 and IL-1β. Therefore, collectively, our study results provide compelling evidence that conventional CD4^+^ T cells that have innate-like features and contribute to autoimmune neuroinflammation and are distinct from previously known innate T cell populations exist.

As our study revealed the heterogeneous characteristics of MP CD4^+^ T cells by single-cell transcriptomic analysis, recent studies have revealed the potential heterogeneity of murine MP CD4^+^ T cells^55–57^. CXCR3^+^T-bet^+^ Th1-like MP CD4^+^ T cells spontaneously generated from naïve CD4^+^ T cells in the steady state show innate-like effector functions against *T. gondii* infection^11^. These cells require DC1-derived tonic IL-12 signaling for optimal differentiation of T-bet^high^ MP T cells^56^. Similarly, we confirmed that CXCR3^high^ MP CD4^+^ T cells, in high correlation with CCR5 expression, could produce IFN-γ and T-bet in response to IL-12 and IL-18, suggesting an innate-like function of CXCR3^high^ MP CD4^+^ T cells. CXCR3^high^ MP CD4^+^ T cells expressed T-bet and were major responders to IL-12 and IL-18 but not IL-23/IL-1β cytokine stimulation (Supplementary Fig. 11a). Similarly, CCR6^high^ MP CD4^+^ T cells were not very responsive to IL-12 and IL-18 (Supplementary Fig. 11b), suggesting that there are distinct responder cells for specific sets of inflammatory cytokines.

We previously demonstrated that IL-23 and IL-1β, which are derived from innate immune cells^29,58–60^, can synergistically increase pathogenic cytokine production in memory-like CD4^+^ T cells in vitro^28^. Additionally, non-myelin-specific CD4^+^ T cells can infiltrate the CNS with MOG antigen-specific T cells, which significantly contributes to EAE disease progression^27,28,61,62^. Consistent with these works, we confirmed that repeated exogenous treatment of mice with IL-23 and IL-1β increased IL-17A and GM-CSF production by MP CD4^+^ T cells (Supplementary Fig. 12). In rheumatoid arthritis patients, T cells that infiltrate the synovial fluid mainly express the memory marker CD45RO and specifically respond to epitopes of Epstein‒Barr virus and cytomegalovirus^31,32,63,64^. In type 1 diabetes, infection with rotavirus or Coxsackie virus is reported to be involved in accelerated diabetes onset through Toll-like receptor (TLR) signaling without pancreatic infection^65,66^, and influenza A virus is linked to diabetes in human patients^67,68^. Collectively, those studies suggest that antigen-nonrelated CD4^+^ T cells can contribute to disease onset or progression with antigen-specific T cells in various autoimmune diseases. Here, we identified CCR6^high^ MP CD4^+^ T cells as the major subpopulation of MP CD4^+^ T cells that respond to IL-23 and IL-1β by expanding and inducing pathogenic Th17 characteristics. In an adoptive transfer model of EAE, IL-23/IL-1β-responsive CCR6^high^ MP CD4^+^ T cells transferred with MOG-specific T cells induced more severe EAE than did CCR6^low^ cells, with increased production of IL-17 and GM-CSF in the CNS. We further confirmed that MP CD4^+^ T cells did not respond to the MOG_33-55_ antigen, indicating the innate-like functions of CCR6^high^ MP CD4^+^ T cells in autoimmune neuroinflammation. In addition, we confirmed that IL-1R1 was required for the pathogenic contribution of CCR6^high^ MP CD4^+^ T cells in EAE (Supplementary Fig. 13), suggesting that IL-1 signaling in CCR6^high^ MP CD4^+^ T cells could trigger their bystander effector functions in vivo. Further studies should be performed to reveal the distinct mechanism connecting antigen-specific T cells and bystander-activated T cells in autoimmune disease pathogenesis.

By analyzing the single-cell transcriptomic data of IL-23/IL-1β-responsive MP CD4^+^ T cells, we identified that Bhlhe40 could be a potential transcriptional regulator inducing GM-CSF expression in CCR6^high^ MP CD4^+^ T cells. Bhlhe40 has been reported to play pivotal roles in T cells. Bhlhe40-deficient naïve CD4^+^ T cells show a limited response to TCR stimulation^69^. In addition, Bhlhe40 seems to be required for Th1 and Th17 effector cytokine production, including IL-17A, GM-CSF and IFN-γ, in the context of autoimmune disease, GVHD, and *Toxoplasma gondii* infection models^29,43–45^. The expression of Bhlhe40 correlates with the mouse *Csf2* locus, which encodes GM-CSF,^43,46^, and a positive correlation with GM-CSF expression was reported in human PBMCs^70^. We demonstrated that steady-state CCR6^high^ MP CD4^+^ T cells expressed RORγt and the IL-1 receptor and upregulated Bhlhe40 in response to IL-23 and IL-1β. In the absence of TCR engagement, they could produce IL-17 and GM-CSF, which importantly contribute to the pathogenesis of EAE. In agreement, a previous study reported that the majority of Bhlhe40-expressing pathogenic T cells in active EAE are not MOG specific^29,43^. As Bhlhe40^–/–^ CCR6^high^ MP CD4^+^ T cells showed reduced GM-CSF production and GM-CSF^–/–^ CCR6^high^ MP CD4^+^ T cells could not exacerbate EAE, Bhlhe40-mediated GM-CSF production seems to be an important mechanism of bystander-activated MP CD4^+^ T cells in EAE. Therefore, Bhlhe40 could be a pivotal transcriptional regulator for both antigen-specific and bystander MP CD4^+^ T cells in the context of CNS inflammation and targeting Bhlhe40 in CD4^+^ T cells may serve as a novel treatment strategy to control autoimmune diseases.

While self-antigen-specific T cells are known to trigger autoimmune inflammation, our findings reveal that antigen-nonrelated steady-state MP CD4^+^ T cells also significantly contribute to pathogenic inflammation in a bystander manner. Our study highlights the importance of considering the bystander function of adaptive immune cells in the context of neuroinflammatory diseases and offers a new avenue for drug development to modulate autoimmune diseases.

## Supplementary information

Supplementary information

## Data Availability

The data that support the findings of this study are available from the corresponding author upon reasonable request. The RNA-seq data have been deposited in the NCBI Gene Expression Omnibus.
