# Supplementary material for: Steady-state memory-phenotype conventional CD4+ T cells exacerbate autoimmune neuroinflammation in a bystander manner via the Bhlhe40/GM-CSF axis
Source: Exp Mol Med. 2023 May 1;55(5):1033–45. doi: 10.1038/s12276-023-00995-1 (PMC10238403; doi:10.1038/s12276-023-00995-1)
Supplement: Supplementary file 1 — Supplementary information [file 12276_2023_995_MOESM1_ESM.pdf]

Supplementary Information

Steady-state memory-phenotype conventional CD4$^+$ T cells exacerbate autoimmune neuroinflammation in a bystander manner via the Bhlhe40/GM-CSF axis

Min-Ji Cho[1,†], Hong-Gyun Lee[1,2,†], Jae-Won Yoon[1], Gil-Ran Kim[1], Ja-Hyun Koo[1,3], Reshma Taneja[4], Brian T. Edelson[5], You Jeong Lee[6], Je-Min Choi[1,7,8,*]

†These authors contributed equally to this work.

[*]Corresponding author. Je-Min Choi Email: jeminchoi@hanyang.ac.kr

Supplementary Methods

Single-cell RNA sequencing sample preparation

MP (TCRβ^{+}CD4^{+}CD25^{-}CD62L^{low}CD44^{high}) CD4^{+} T cells from the spleens of 10-week-old mice were isolated using a FACS Aria cell sorter II (BD Biosciences, Franklin Lakes, NJ, USA). FACS-sorted MP CD4^{+} T cells were stimulated with IL-1β (20 ng/mL, R&D Systems, Minneapolis, MN, USA), IL-23 (20 ng/mL, R&D Systems), IL-12 (20 ng/mL, Peprotech, Rocky Hill, NJ, USA), IL-18 (20 ng/mL, R&D Systems), IL-33 (20 ng/mL, R&D Systems), IL-25 (20 ng/mL, R&D Systems), or IL-7 (10 ng/mL, Peprotech, Rocky Hill, NJ, USA) for 5 days at 37 °C in an incubator.

Single-cell RNA- and TCR-seq data processing

Raw sequencing data were processed using a well-developed program, Cellranger (10X genomics), for quality control (QC) and gene expression estimation. Low-quality cells were filtered using 4 QC metrics: the percentage of mitochondrial gene unique molecular identifier (UMI) counts, the number of expressed genes (UMI count > 0), the sum of UMI counts per cell, and the doublet score estimated using Doublet Finder [10.1016/j.cels.2019.03.003]. The outlier cells from each QC metric were excluded from further analyses. To collect pure TCRα/β^{+} CD4^{+} T cells, cells expressing PLZF (*Zbtb16*) and *Cd74*$^{+}$-cells in which at least 1 UMI was counted were removed. All TCRα/β^{+} CD4^{+}T cells were integrated by cytokine treatment (set 1: IL-7, IL-7+ IL-1β, and IL-7+ IL-1β +IL-23; set 2: IL7, IL7+IL-25, IL-7+IL-33, and IL-7+IL-25+IL-33; set 3: IL-7, IL-7+IL-12, IL-7+IL-18, and IL-7+IL-12+IL-18) using monocle3 [10.1038/nmeth.4402]. TCR-seq raw sequencing data were processed using the Cellranger 'adj' program. The TCR clonotypes from previously filtered scRNA-seq were used in the further analyses.

Single-cell RNA-seq data analysis

The preprocessed scRNA-seq data were aligned with the IL-7 treatment group using 'align_cds' from the monocle3 program [https://doi.org/10.1038/s41586-019-0969-x], and data were integrated for each set. Each cluster was characterized using the marker gene expression levels and referring to previous studies. The top specifically expressed genes of each cluster were selected by the specificity of each gene, as estimated using 'FindAllMarkers' in Seurat v4.0 [https://doi.org/10.1016/j.cell.2021.04.048], and then their expression level was visualized using 'DoHeatmap' in the same program. We calculated the percentage of cells in each condition for each cell type by dividing the number of cells in each condition by the number of clustered cells. A DEGs analysis was performed for each cytokine treatment of each set using 'FindMarkers' in Seurat with the following parameters: min.pct = 0, min.cells.feature = 1, min.cells.group = 1, logfc.threshold = 0. DEGs between cytokine treatments and DEGs between conditions were defined at FDR <0.05 and average log2 fold change ≥0.1. A functional analysis of DEGs and cluster specific gene sets was performed in MSigDB's C2 (KEGG) and C5 (GO). The functions were analyzed using the maximum and minimum size of the gene sets (KEGG with min 2 genes and max 500, GO with min 2 genes and max 150 genes). The gene set enrichment test was performed

using the 'enricher' function of the clusterProfile package [10.1089/omi.2011.0118]. The significance level was set as the function that satisfied FDR < 0.2 and had at least 2 overlapping genes.

Signature score of the MP CD4$^+$ T cells

The signature score of the CD4$^+$ T cells was estimated to define the features of each cell type. The monocle3 object of each set of cytokine treatments was transformed into the Seurat object. They were normalized by condition and then integrated. The signature score of each cell type was calculated using AddModuleScore in the Seurat package.

Trajectory inference of MP CD4$^+$ T cells by cytokine stimulation

Effector memory CD4$^+$ T-cells have the plasticity to differentiate into various helper cells. We performed a trajectory analysis on the subcluster composed of the initial cells that are likely to be differentiated according to each cytokine stimulus and the cells that responded. First, we selected 2,000 highly variable genes by cell type, and then we inferred the trajectory using reduceDimension with DDRTree in the monocle2 program [10.1038/nbt.2859]. Pseudo-time was calculate using orderCells in the same program. DEGs following the pseudo-time trajectory were defined at a q-value < 1e-50.

Estimation of regulon activity score

Regulon describes the relationship between a transcription factor and its regulatory target genes, and its activity is calculated using the expression levels of the genes in the regulon. First, we constructed a correlation matrix between genes, and then we calculated the activity of each regulon using GENIE3 [10.1371/journal.pone.0012776]. At that time, all the parameters were set to the default. The regulon relationship was derived from mm10__refseq-r80__500bp_up_and_100bp_down_tss.mc9nr and mm10__refseq-r80__10kb_up_and_down_tss.mc9nr in the ‘cisTarget_databases’ of the R package. All processes were performed using the SCENIC program in R [10.1038/nmeth.4463]. Differentially activated regulons were considered only when they contained at least 1% of expressing cells.

T-cell receptor repertoire analysis

The TCR repertoire of the cells was considered only from the scRNA-seq data. The degree of TCR expansion was categorized as hyperexpanded (100 < X <= 500), large (20 < X <= 100), medium (5 < X <= 20), small (1 < X <= 5), and single (0 < X <= 1). The TCR diversity of each sample was calculated using Shannon’s index, which considers non-uniformity in the frequency of the clonotype (gene and nucleotide sequences). All procedures were performed using scRepertoire [10.12688/f1000research.22139.2] in R.

Supplementary Figures

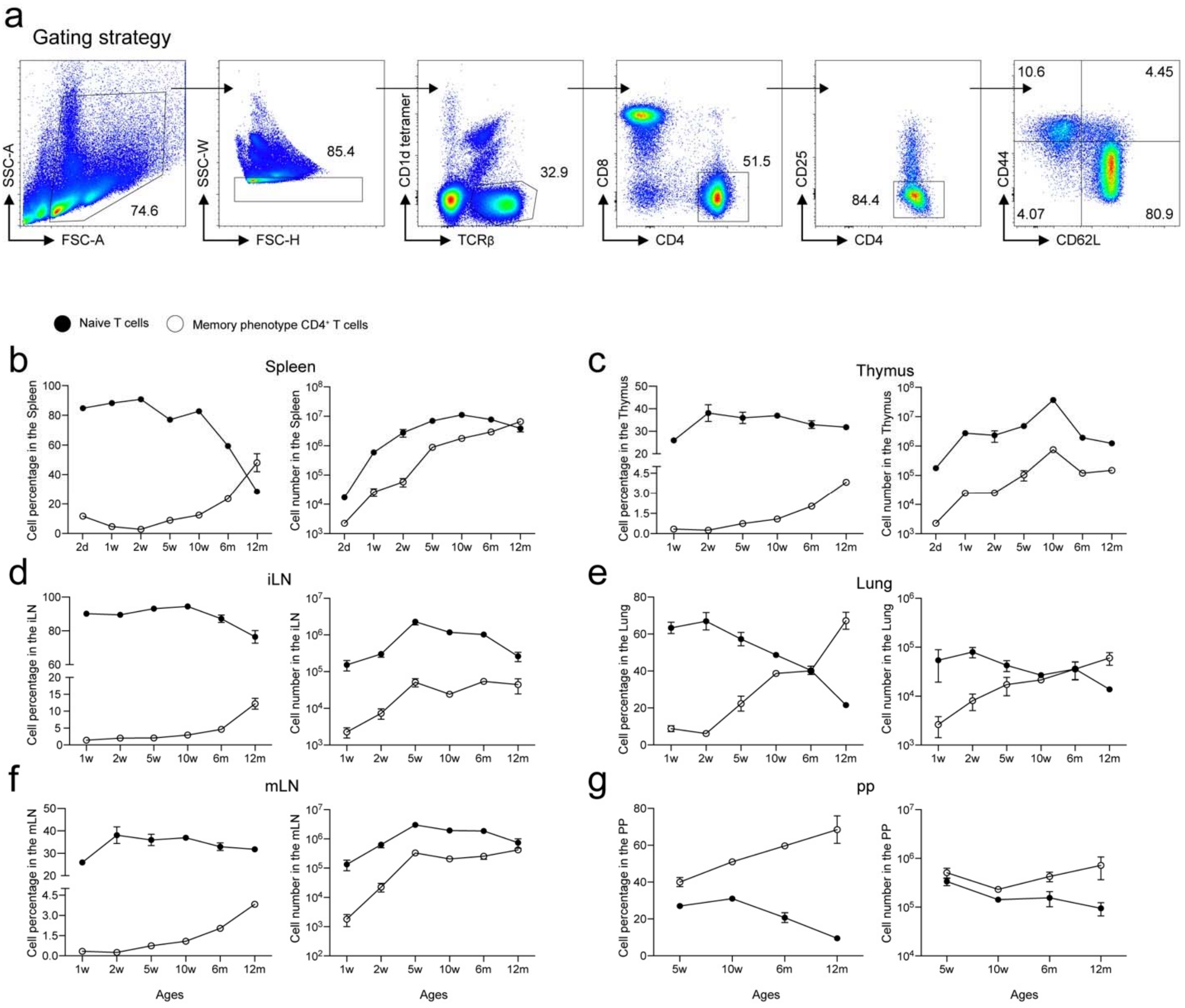

Supplementary Fig. 1 Steady state CD44^{high} memory phenotype (MP) CD4^{+} T cells increase with age in mice. (a) Gating strategy of Splenic MP CD4^{+} T cells (TCRβ^{+}CD1d tetramer^{-}CD8^{-}CD4^{+}CD25^{-}CD62L^{low}CD44^{high}). Analysis of cell percentage and number of naïve (TCRβ^{+}CD1d tetramer^{-}CD8^{-}CD4^{+}CD25^{-}CD62L^{high}CD44^{low}) and MP CD4^{+} T cells (TCRβ^{+}CD1d tetramer^{-}CD8^{-}CD4^{+}CD25^{-}CD62L^{low}CD44^{high}) by age from (b) the spleen, (c) thymus, (d) iLN, (e) lung, (f) mLN, and (g) PP.

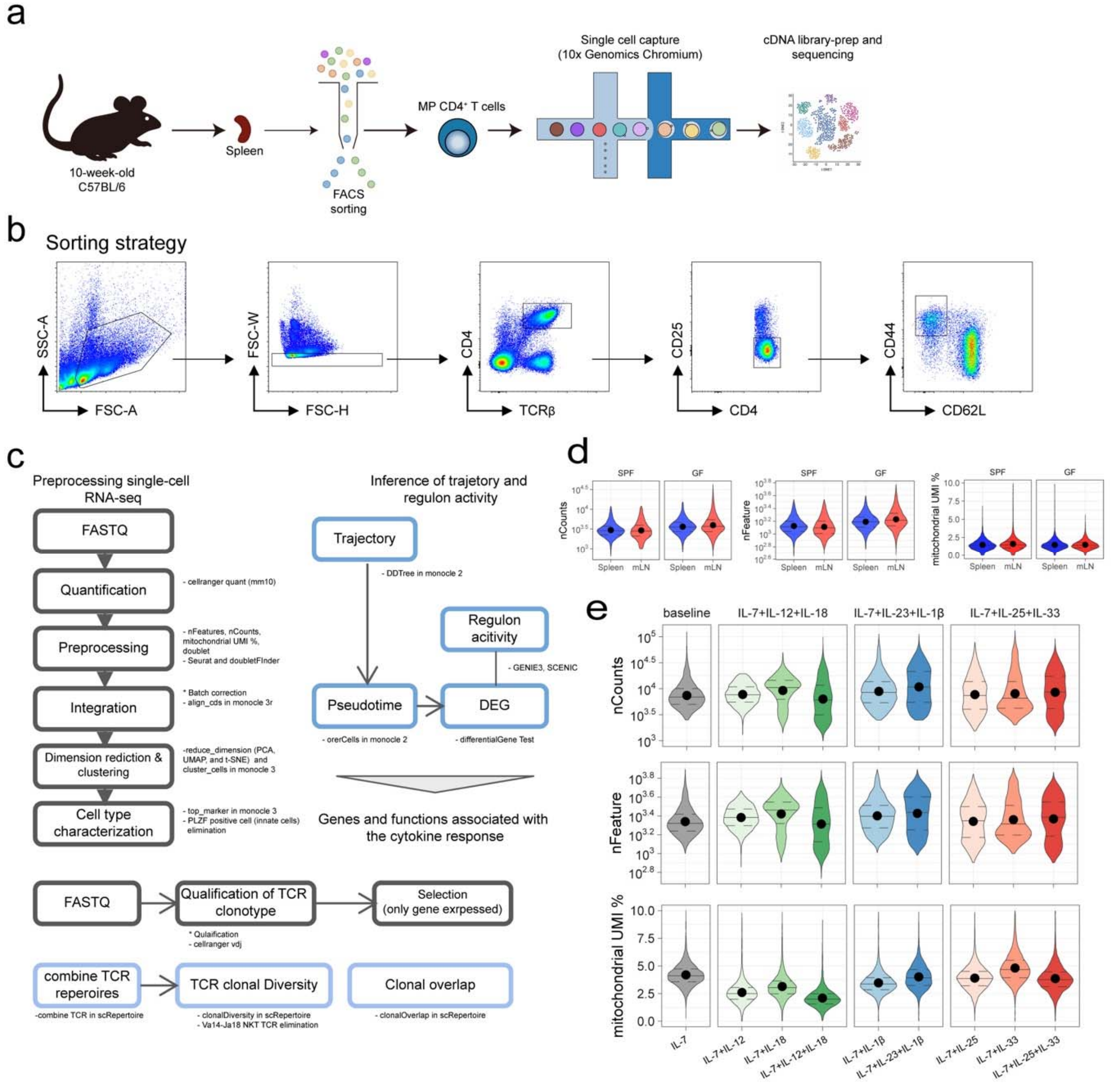

83

Supplementary Fig. 2 Strategy and flow of single cell RNA-sequencing analysis. (a) Visual concept of scRNA-seq for MP CD4$^+$ T cells. (b) Gating strategy of splenic MP (TCRβ$^+$CD4$^+$CD25$^-$CD62L^{low}CD44^{high}) CD4$^+$ T cells. (c) Workflow of scRNA-seq and TCR-seq of PLZF and TCR V$_{\alpha}$14-J$_{\alpha}$18 (TRAV11-TRAJ18) negative MP CD4$^+$ T cells. (d) Quality measures of splenic and mLN MP CD4$^+$ T cells from SPF- and GF–housed mice. (e) QC metrics for each condition of MP CD4$^+$ T cells. (nFeature: the number of expressed genes per cell, nCounts: the number of total UMI counts per cell, mitochondrial UMI %: the percentage of UMI counts of mitochondrial genes per cell)

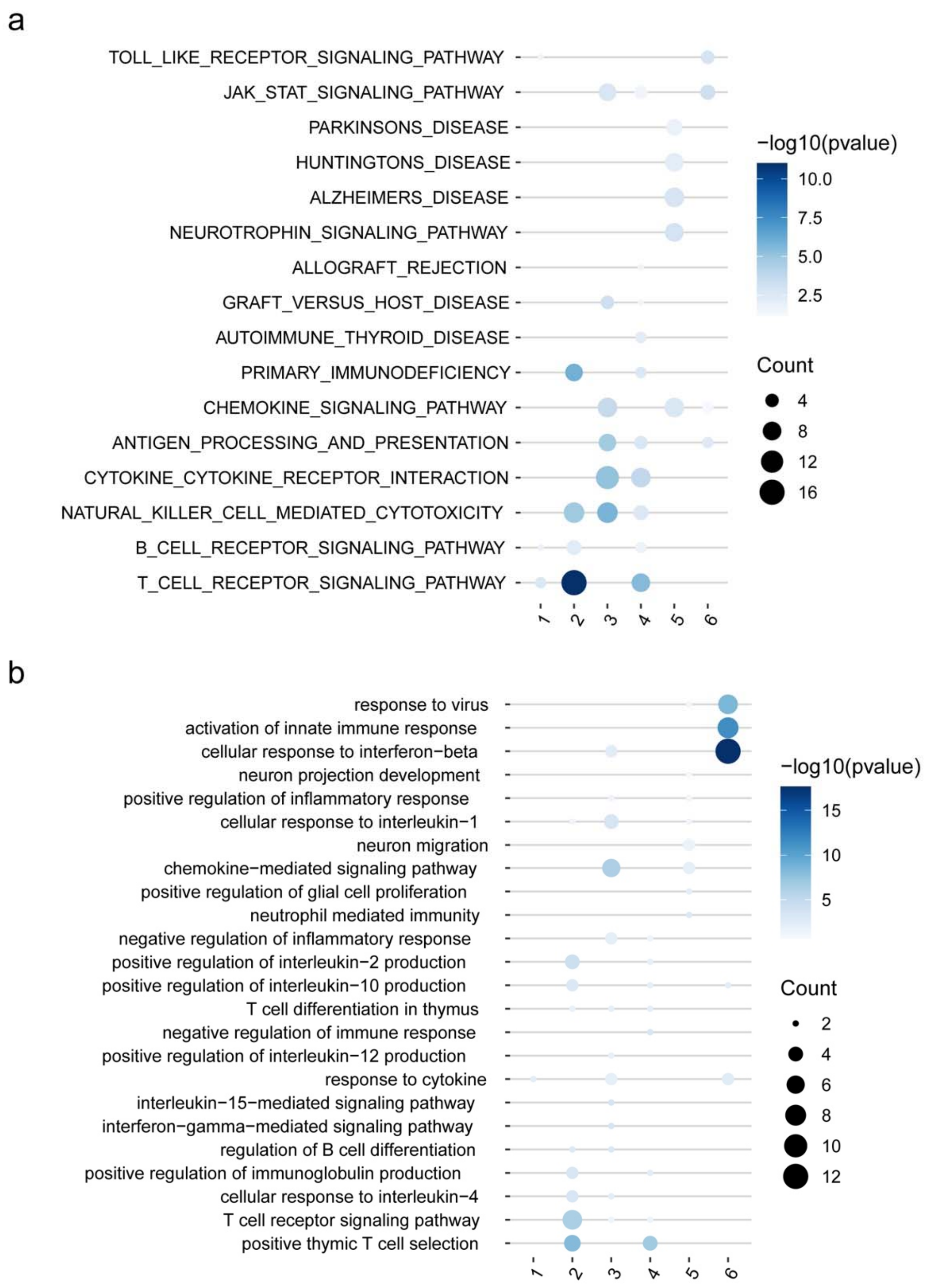

Supplementary Fig. 3 KEGG/GO analysis of steady-state MP CD4$^+$ T cells. Selected (a) KEGG and (b) GO terms in each subpopulation of splenic MP CD4$^+$ T cells.

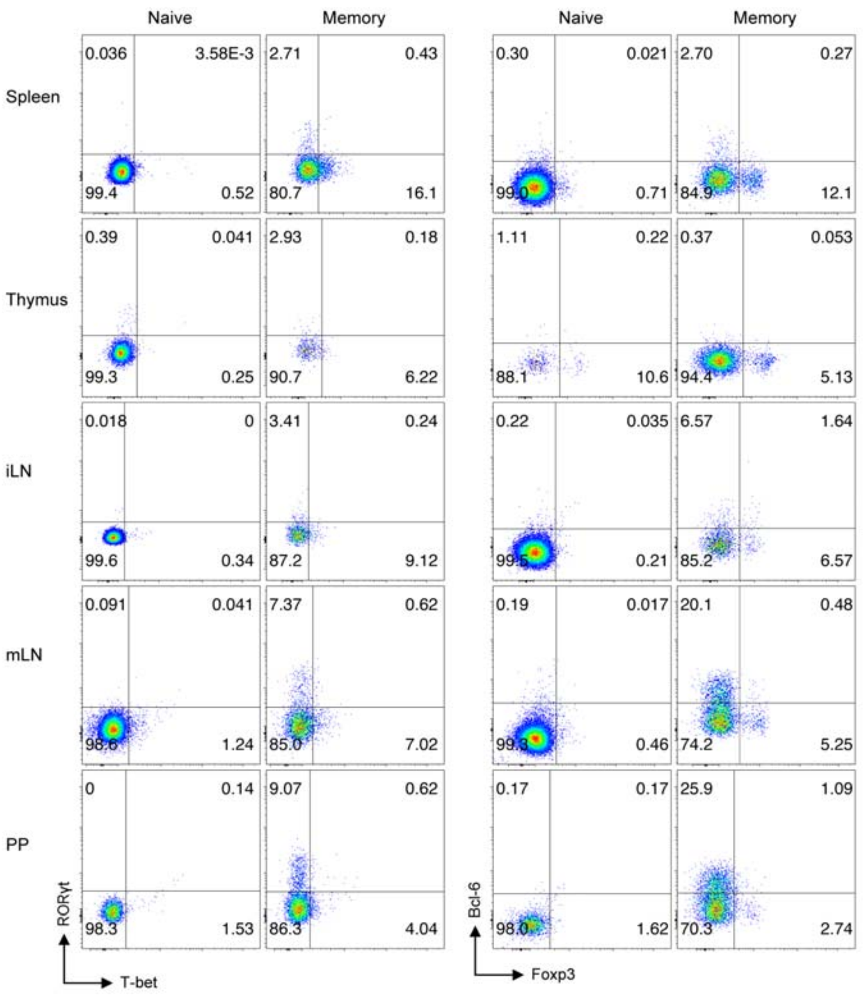

Supplementary Fig. 4 Effector T cell lineage–specific marker expression in MP CD4$^+$ T cells. Transcription factor expression levels of T-bet, RORγt, Bcl-6, and Foxp3 in naïve and MP CD4$^+$ T cells from the spleen, thymus, iLN, mLN, and PP.

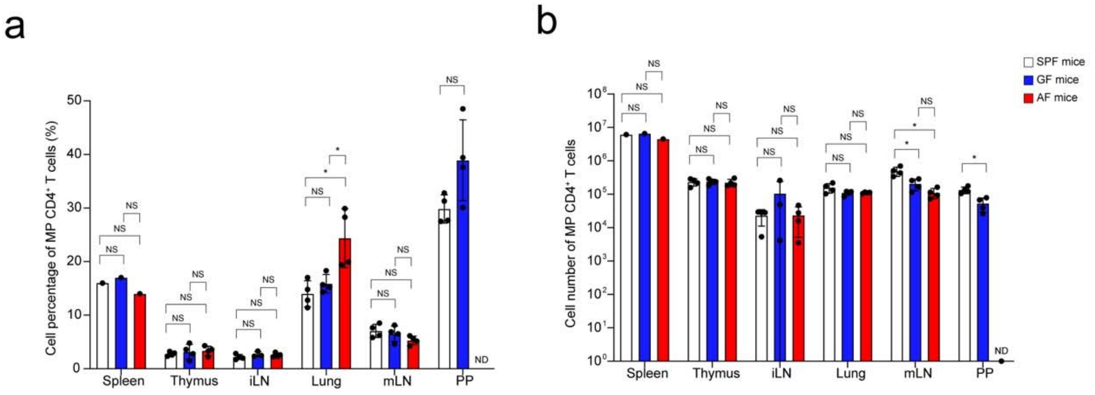

Supplementary Fig. 5 Comparison of MP CD4$^+$ T cells in tissues from SPF, GF, and AF mice. (a) Proportion and (b) number of MP CD4$^+$ T cells in the spleen, thymus, iLN, lung, mLN, and PP in SPF, GF, and AF mice (n=4). Data are presented as the mean ± S.D. P values were calculated using Mann-Whitney U-test (ND, not detected; NS, not significant; *$p < 0.05$, **$p < 0.01$, ***$p < 0.001$).

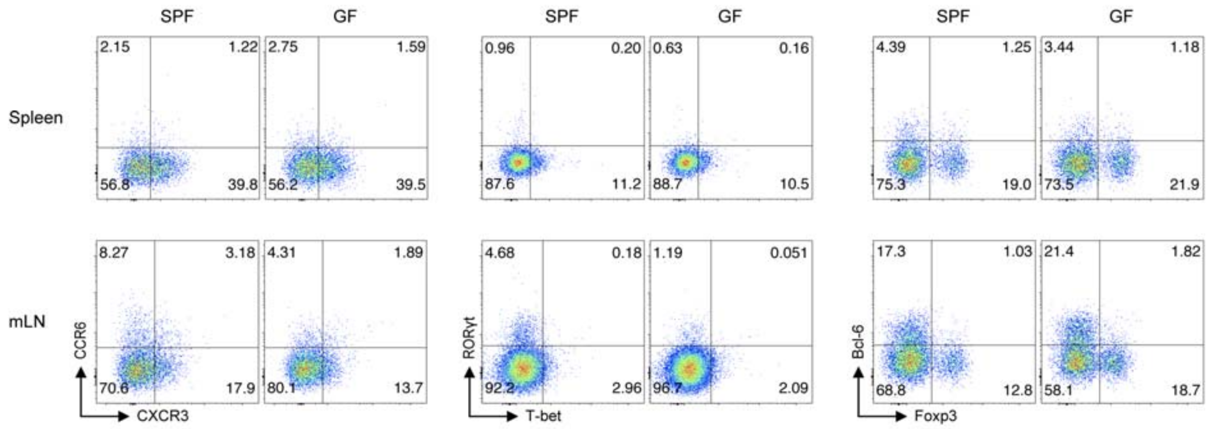

Supplementary Fig. 6 Effector T cell lineage–specific chemokine receptors and transcription factors in MP $CD4^+$ T cells from SPF and GF mice. Expression of chemokine receptor of CCR6, CXCR3 and transcription factors of T-bet, RORγt, Bcl-6, and Foxp3 in spleen- and mLN-derived MP $CD4^+$ T cells from SPF and GF mice.

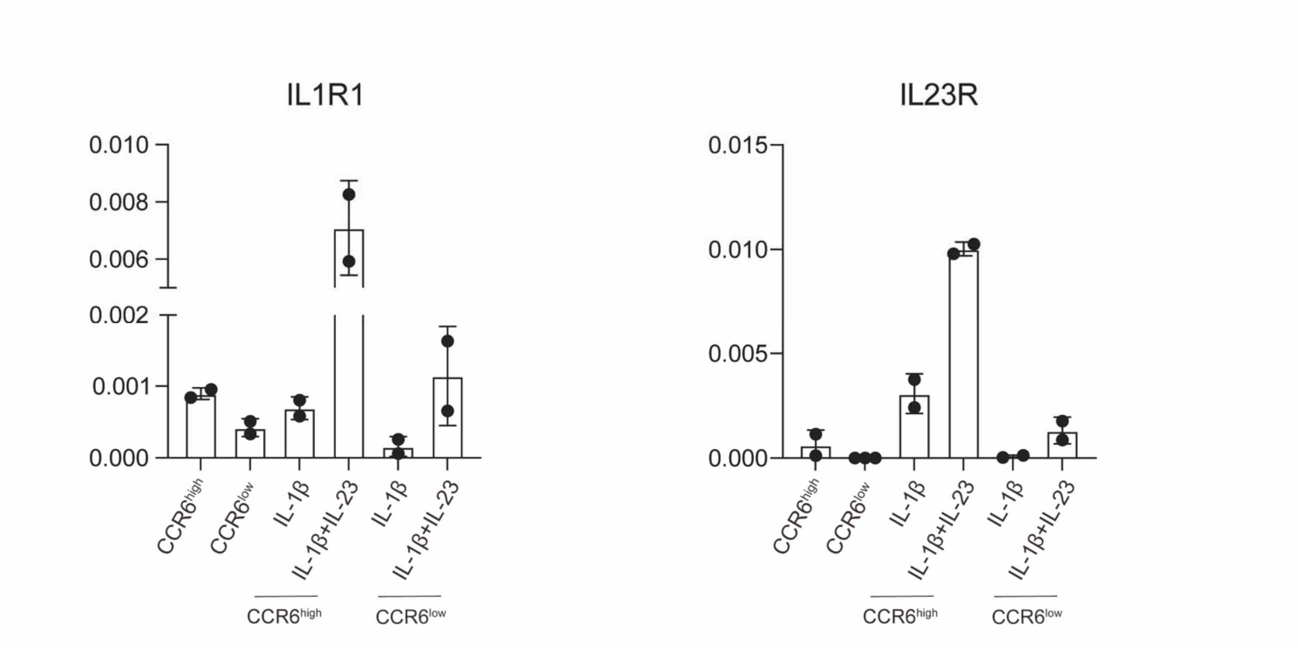

Supplementary Fig. 7 IL1R1 and IL23R RNA expression level of CCR6^{high} and CCR6^{low} MP CD4^{+} T cells. mRNA expression of IL-1β and IL-23 receptors in steady-state CCR6^{high} and CCR6^{low} MP CD4^{+} T cells and in the cells after activation with single or combination treatment of IL-1β/IL-23 (n=2).

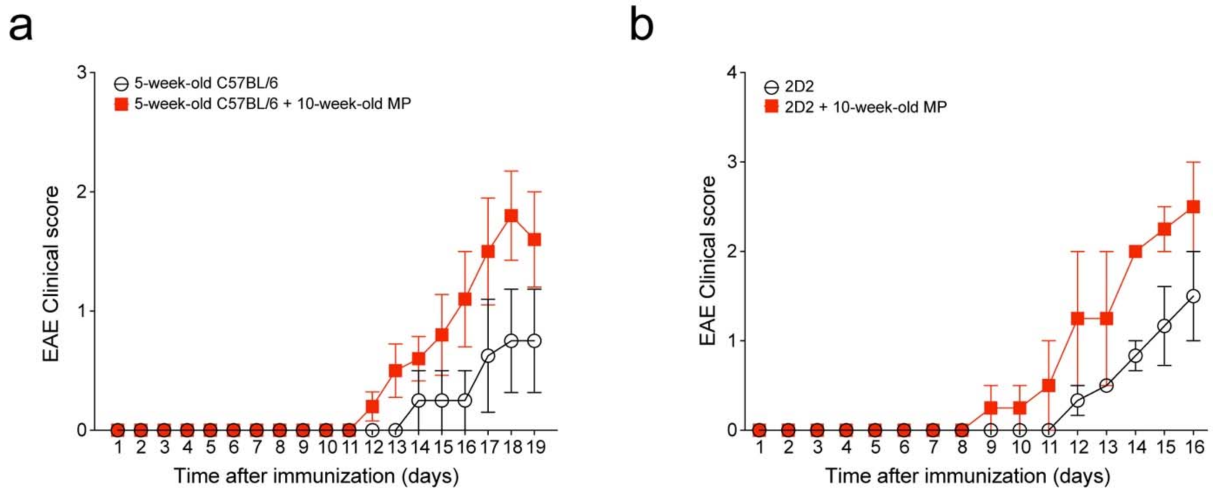

Supplementary Fig. 8 Addition of MP CD4$^+$ T cells in mice exacerbate autoimmune encephalomyelitis. (a) 5-week-old C57BL/6 mice with or without adoptively transferred 5 × 10^5 Treg-deleted MP CD4$^+$ T cells (TCRβ$^+$CD1d tetramer$^-$CD4$^+$Foxp3$^-$CD62L^{low}CD44^{high}) from 10-week-old Foxp3-GFP mice were induced active EAE by $MOG_{35\text{-}55}$ in CFA (n=4). (b) 5 × 10^4 naïve CD4$^+$ T cells (TCRβ$^+$CD4$^+$$V_\beta$11$^+$CD25$^-$CD62L^{high}CD44^{low}) from 2D2 transgenic mice were adoptively transferred, with or without 5 ×10^5 splenic Treg-deleted MP CD4$^+$ T cells (CD45.1$^+$γδTCR$^-$NK1.1$^-$$V_\beta$11$^-$TCRβ$^+$CD4$^+$CD1d tetramer$^-$CD25$^-$CD62L^{low}CD44^{high}) sorted from Foxp3-GFP mice, into Rag$^{-/-}$ mice who were immunized with $MOG_{35\text{-}55}$ in CFA (n = 3).

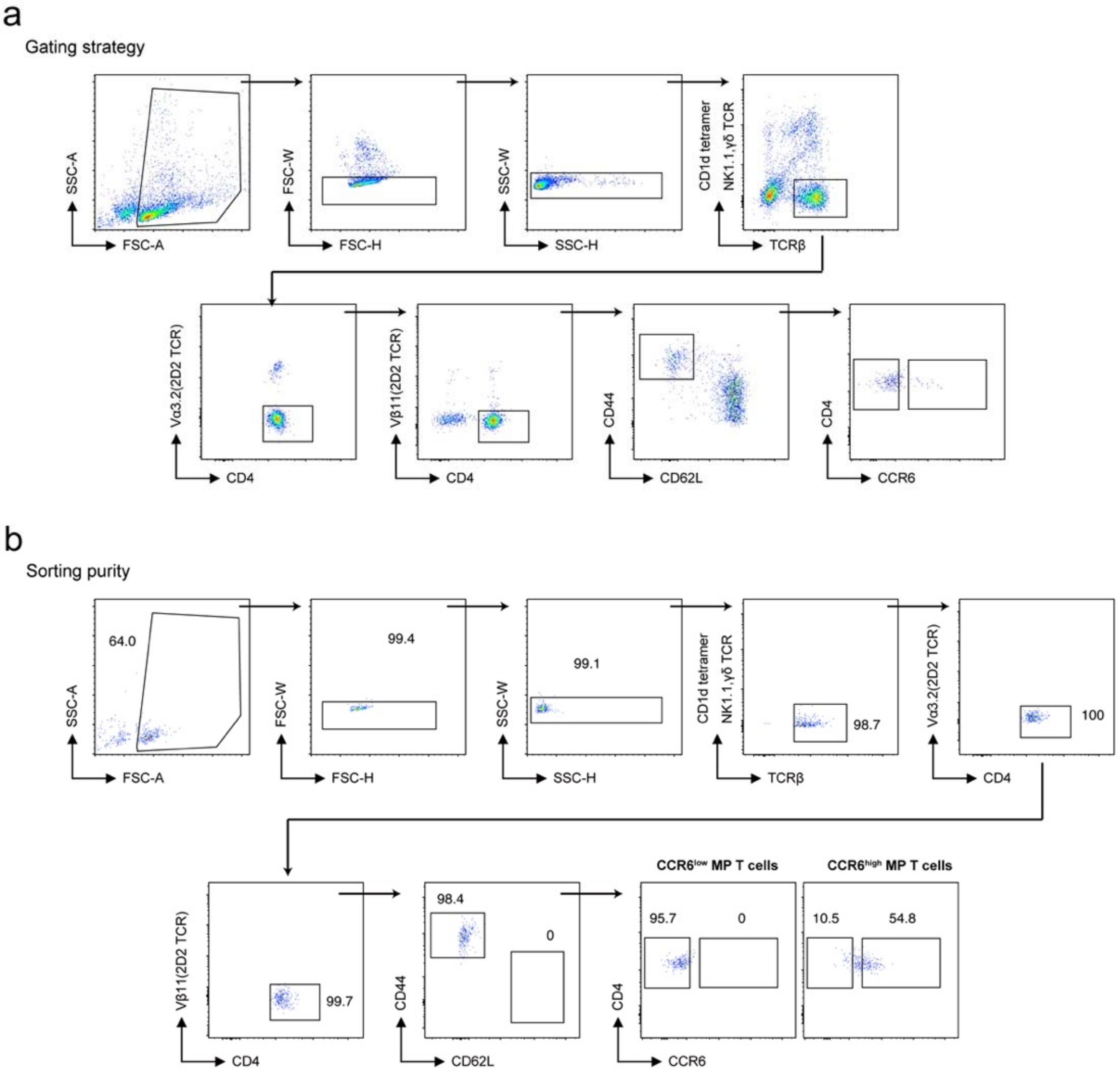

Supplementary Fig. 9 Gating strategy and sorting purity of CCR6^{high} and CCR6^{low} MP CD4$^+$ T cells. (a) Gating strategy of splenic CCR6^{high} and CCR6^{low} MP CD4$^+$ T cells. (b) Sorting purity of splenic CCR6^{high} and CCR6^{low} MP CD4$^+$ T cells.

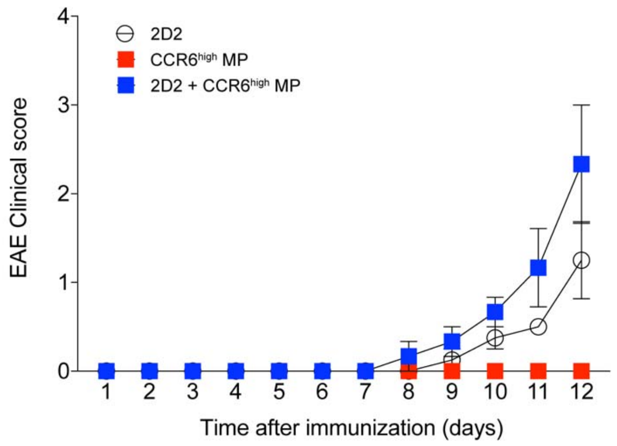

98

Supplementary Fig. 10 CCR6^{high} MP CD4^{+} T cells alone could not induce EAE disease. Naïve CD4^{+} T cells (5 × 10^{4}) from 2D2 transgenic mice were adoptively transferred, with or without CCR6^{high} or CCR6^{high} MP CD4^{+} T cells alone (1 × 10^{5}, CD45.1^{+}γδTCR^{-}NK1.1^{-}V$_{β}$11^{-}TCRβ^{+}CD4^{+}CD1d tetramer^{-}CD25^{-}CD62L^{low}CD44^{high}) of WT mice, into Rag$^{-/-}$ mice who were immunized with MOG$_{35-55}$ in CFA. EAE clinical score was monitored daily (n=3).

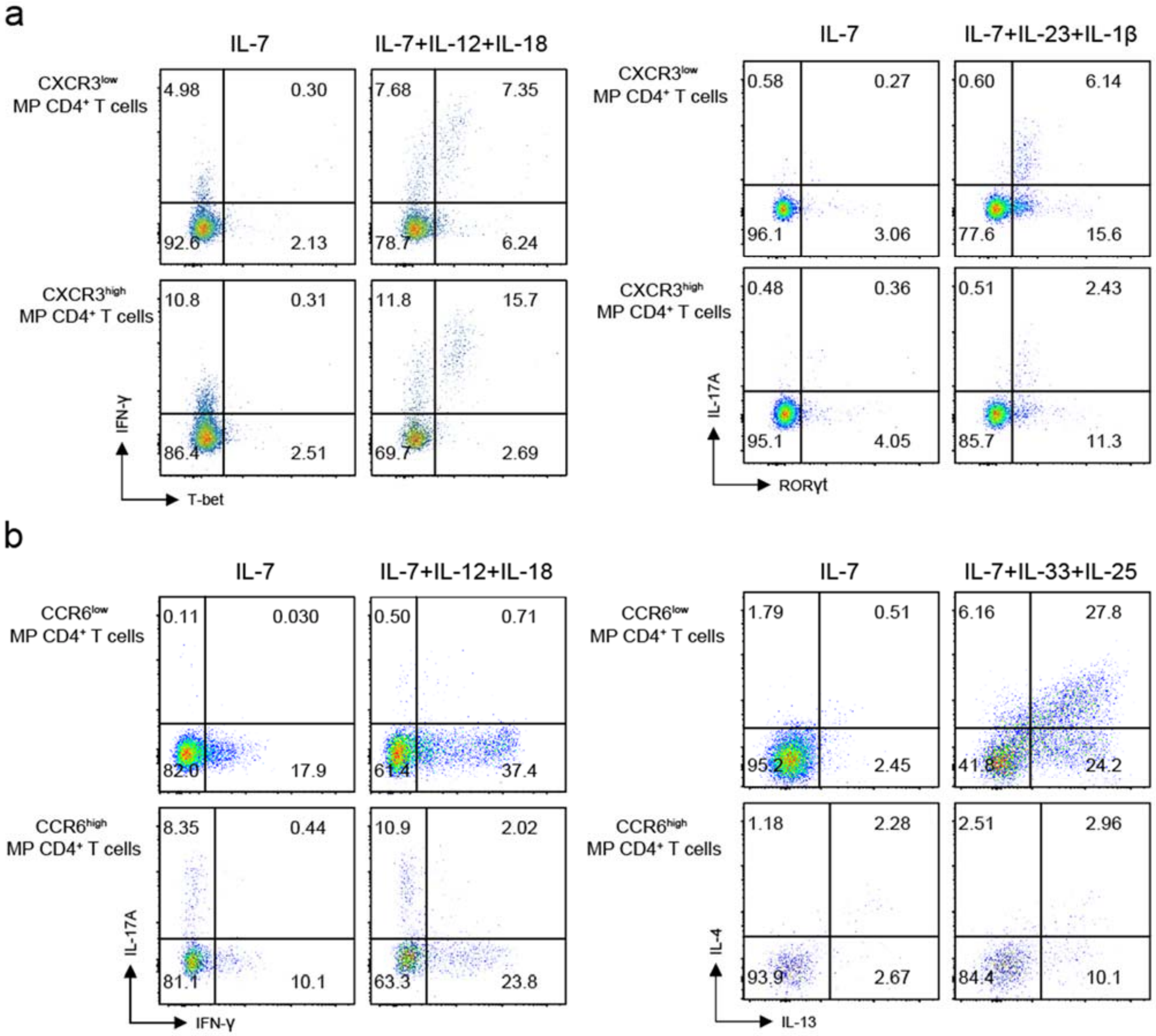

Supplementary Fig. 11 Specific response pattern by MP CD4$^+$ T cell subsets to the cytokines. (a) FACS-sorted CXCR3^{high} and CXCR3^{low} MP CD4$^+$ T cells were stimulated with IL-12/IL-18 and IL-1β/IL-23 in the presence of IL-7 for 5 days. (b) FACS-sorted CCR6^{high} and CCR6^{low} MP CD4$^+$ T cells were stimulated with IL-12/IL-18 and IL-25/IL-33 in the presence of IL-7 for 5 days.

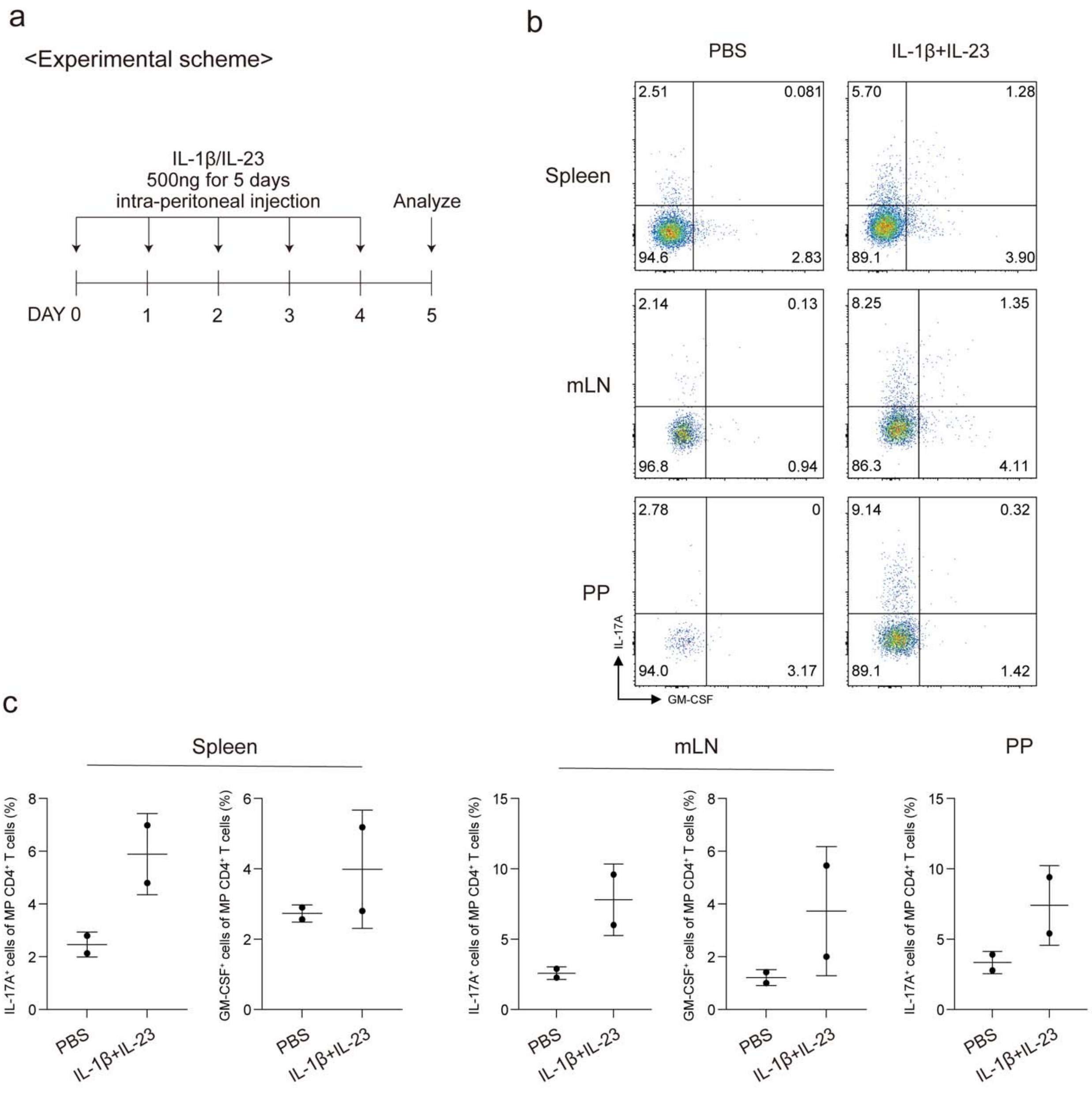

Supplementary Fig. 12 In vivo production of IL-17A and GM-CSF by MP CD4$^+$ T cells upon exogenous IL-1β and IL-23 treatment. (a) Graphical experimental scheme. (b) Representative dot plots showing the population of IL-17A and GM-CSF producing memory-phenotype (MP) CD4$^+$ T cells (TCRβ$^+$CD1d tetramer$^-$CD8$^-$CD4$^+$CD25$^-$CD62L^{low}CD44^{high}) in murine spleens, mesenteric lymph nodes(mLNs), and Payer's patches (PP). (c) The proportion of IL-17A$^+$ and GM-CSF$^+$ cells in MP CD4$^+$ T cells (n=2).

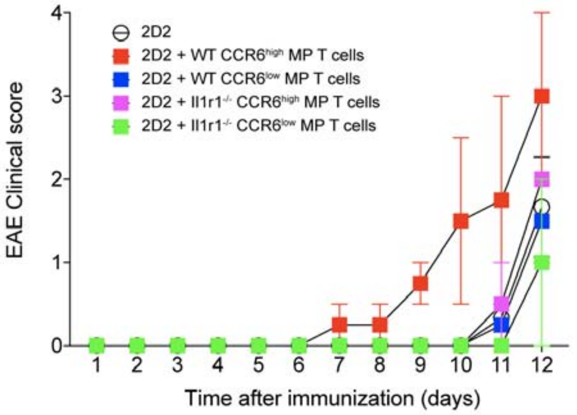

Supporting Fig. 13 IL-1R is required for CCR6^{high} MP CD4^{+} T cells to exacerbate autoimmune encephalomyelitis. Naïve CD4^{+} T cells (5 × 10^{4}) from 2D2 transgenic mice were adoptively transferred, with or without CCR6^{high} or CCR6^{low} MP CD4^{+} T cells (1 × 10^{5}, CD45.1^{+}γδTCR^{-}NK1.1^{-}V$_{\beta}$11^{-}TCRβ^{+}CD4^{+}CD1d tetramer^{-}CD25^{-}CD62L^{low}CD44^{high}) of WT or Il1r1$^{-/-}$ mice, into Rag$^{-/-}$ mice who were immunized with MOG$_{35-55}$ in CFA. EAE clinical score was monitored daily (n=4).
